# Analysis of Indole Alkaloids from *Rhazya stricta* Hairy Roots by Ultra-Performance Liquid Chromatography-Mass Spectrometry

**DOI:** 10.3390/molecules201219873

**Published:** 2015-12-17

**Authors:** Amir Akhgari, Into Laakso, Tuulikki Seppänen-Laakso, Teijo Yrjönen, Heikki Vuorela, Kirsi-Marja Oksman-Caldentey, Heiko Rischer

**Affiliations:** 1 VTT Technical Research Centre of Finland Ltd., P.O. Box 1000, FI-02044 VTT, Finland; amirakhgari1357@gmail.com (A.A.); tuulikki.seppanen-laakso@vtt.fi (T.S.-L.); kirsi-marja.oksman@vtt.fi (K.-M.O.-C.); 2 Division of Pharmaceutical Biosciences, Faculty of Pharmacy, P.O. Box 56, FI-00014 University of Helsinki, Finland; into.laakso@helsinki.fi (I.L.); teijo.yrjonen@helsinki.fi (T.Y.); heikki.vuorela@helsinki.fi (H.V.)

**Keywords:** *Rhazya stricta*, terpenoid indole alkaloids, ultra-performance liquid chromatography-mass spectrometry

## Abstract

*Rhazya stricta* Decne. (Apocynaceae) contains a large number of terpenoid indole alkaloids (TIAs). This study focused on the composition of alkaloids obtained from transformed hairy root cultures of *R. stricta* employing ultra-performance liquid chromatography-mass spectrometry (UPLC-MS). In the UPLC-MS analyses, a total of 20 TIAs were identified from crude extracts. Eburenine and vincanine were the main alkaloids followed by polar glucoalkaloids, strictosidine lactam and strictosidine. Secodine-type alkaloids, tetrahydrosecodinol, tetrahydro- and dihydrosecodine were detected too. The occurrence of tetrahydrosecodinol was confirmed for the first time for *R. stricta*. Furthermore, two isomers of yohimbine, serpentine and vallesiachotamine were identified. The study shows that a characteristic pattern of biosynthetically related TIAs can be monitored in *Rhazya* hairy root crude extract by this chromatographic method.

## 1. Introduction

*Rhazya stricta* Decne. belongs to the Apocynaceae family and is widely distributed in the Middle East and Indian sub-continent. The plant has a long history in folk medicine and has been used to treat several diseases [1]. It produces a large number of terpenoid indole alkaloids (TIAs) classified into 17 subgroups. Hitherto, more than 100 alkaloids have been found in *R. stricta* [2].

Structure elucidation of isolated *Rhazya* alkaloids has been carried out by NMR spectroscopy and high-resolution mass spectrometry (HRMS) operated in electron ionization (EI) mode [1]. A reversed-phase high-performance liquid chromatography (RP-HPLC) method has been developed for the separation of alkaloids from somatic hybrid cell cultures of *Rauwolfia serpentina* × *R. stricta* using phosphate buffer and ion pair reagent [3]. It is a common practice to use standard compounds in the identification of alkaloids in HPLC-UV-MS, since the chromatographic and spectroscopic properties of standard compounds can be compared with those present in the extract. However, the availability of authentic standard compounds is limited since only a few pure compounds present in *R. stricta* are commercially available and components must therefore be isolated and purified from crude extracts, which makes the identification complex and time consuming [4]. Recently, an improved chromatographic separation of alkaloids was achieved by the introduction of ultra-performance liquid chromatography (UPLC) [5,6,7]. In UPLC, the stationary phase particle size of less than 2.5 µm has been shown to result in a significant improvement in separation efficiency [8].

In our previous study, we established hairy root cultures of *R. stricta* and investigated the accumulation of major alkaloids in transgenic roots by HPLC. The identification of the alkaloids was based on the correlation of UV and MS data from HPLC and UPLC analyses [9]. *Rhazya* alkaloids comprise a wide range of structures and polarities; therefore, it was necessary to first develop analytical methods to determine the alkaloids. Using gas chromatography-mass spectrometry (GC-MS), a total of 20 compounds, including six new compounds for *Rhazya*, mainly non-polar alkaloids, were found on *Rhazya* alkaloids in our previous study [10]. The present work particularly aims at in depth identification of more polar alkaloids in crude extracts of hairy roots by ultra-performance liquid chromatography-mass spectrometry (UPLC-MS) analysis that could not be analysed by GC-MS.

## 2. Results

### 2.1. UPLC-Photodiode Array (PDA) Method Test

Two isocratic and three gradient programs were carried out. The first isocratic run, containing 55% solvent A (10 mM ammonium acetate, pH 10 in water), showed better separation compared to the second run, but major peaks still overlapped during the first three minutes. Therefore, smaller compounds were separated better. In the second isocratic run, with 65% solvent A, the majority of the compounds were eluting very rapidly, even before three minutes.

In the first gradient run, containing 0.1% formic acid in water (solvent A), all the major peaks eluted in the first two minutes. In the second gradient run, with 1% formic acid, several minor components co-eluted with three major peaks in the middle of the run. The best separation of these experiments was obtained in the third gradient run (solvent A, pH 10/acetonitrile solvent B), and these conditions were used in the UPLC-PDA-MS analyses of three representative wild type hairy root clones 2, 3 and 10.

### 2.2. Alkaloid Composition 

#### 2.2.1. Analysis of Reference Substances by UPLC-PDA-MS

Four reference substances were analysed in positive ion mode (Table 1). A total ion scan of vincanine showed a protonated molecular ion ([M + H]^+^) at *m*/*z* 293. Other major fragments were not detected. Yohimbine had three UV maxima and an [M + H]^+^ ion at *m*/*z* 355 accompanied by three minor fragments. Vincamine, having a similar UV spectrum and molecular weight as yohimbine, could be distinguished from it by an intense fragment ion *m*/*z* 337, due to the loss of water ([M + H − H_2_O]^+^). Tabersonine displayed three UV maxima. The molecular ion of this compound was detected at *m*/*z* 337, together with a smaller fragment at *m*/*z* 305 due to the loss of methanol ([(M + H − CH_3_OH)]^+^).

**Table 1:** UPLC-PDA-MS data for reference compounds in positive ion mode.

Pure Substance	t_R_ (min)	UV (nm) λ_max_	MW	[M + H]^+^ *m*/*z*	Fragments *m*/*z* (rel. int. %)
Vincanine	2.45	246, 300, 365	292	293 (100)	-
Yohimbine	3.34	227, 279	354	355 (100)	212 (18), 144 (20), 224 (5)
Vincamine	5.02	228, 280	354	355 (100)	337 (90)
Tabersonine	19.19	229, 298, 330	336	337 (100)	305 (50)

#### 2.2.2. Identification of Alkaloids by UPLC-PDA-MS

**Figure 1:**
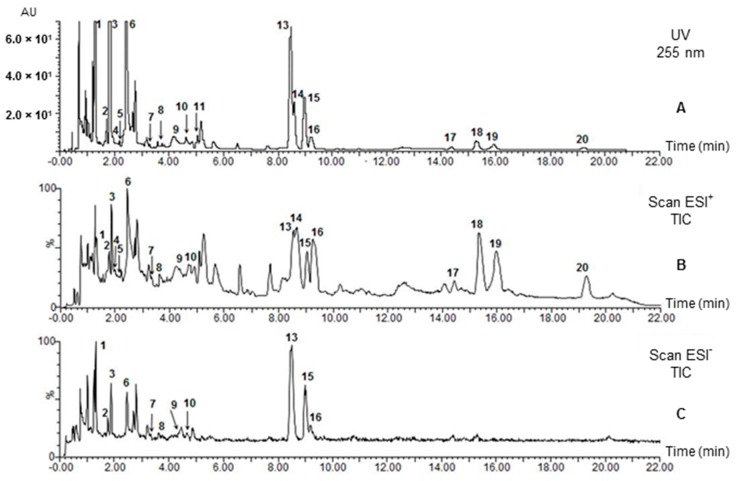
UPLC-UV chromatogram (**A**) and MS-ESI^+^ (**B**) and -ESI^−^ (**C**) iongrams of *R. stricta* alkaloids. Data for vincadifformine (**12**) is presented in Figure S3.

The UPLC-UV chromatogram of a *Rhazya* extract at 255 nm is illustrated in Figure 1A. Comparisons between electrospray ionization (ESI) techniques demonstrate that the majority of abundant alkaloids could be detected in positive ion mode (ESI^+^) (Figure 1B). Negative ion analyses (ESI^−^) exhibited clearly fewer peaks, but instead, cleaner spectra were obtained. Practically no proper peaks were observed after 10 min of elution (Figure 1C). The formation of sodium adducts ([M + H + Na]^+^) in positive ion mode and acetate adducts [M − H + CH_3_COOH]^−^ in negative ion mode was observed for some compounds (Table 2). The UV and mass spectra of the target alkaloids have been collected and presented in Figures S1 and S2, respectively.

**Figure 2:**
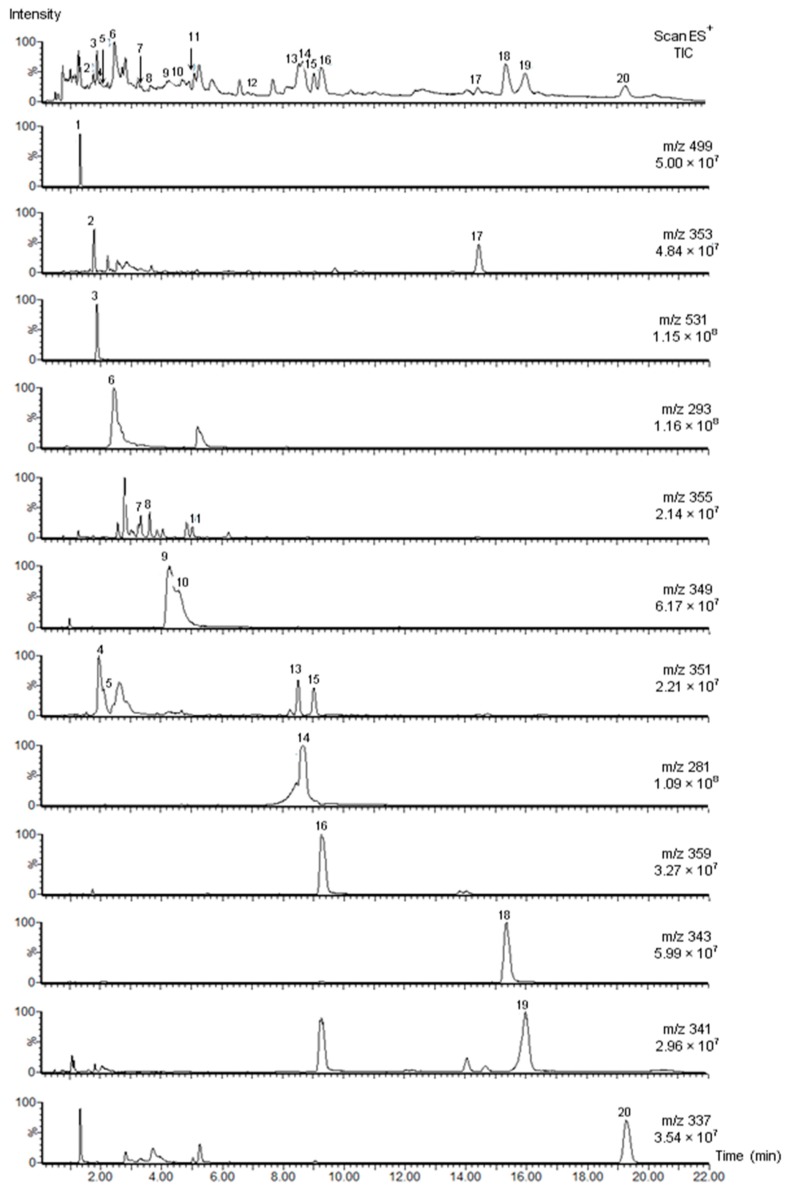
Extracted iongrams (ESI^+^) of UPLC-PDA-MS-total ion current (TIC) analysis of *R. stricta* alkaloids. Data for vincadifformine (**12**) is presented in Figure S3.

It also became evident from the total ion scan (Figure 1B) that baseline separations could not be obtained. A number of symmetric peaks practically without extra signals were detected using extracted ion recording. This method was also used to detect the compounds with the same molecular ion (Figure 2). The alkaloids were identified based on their UV spectra, [M + H]^+^ and [M − H]^−^ ions and fragment ions, the data from reference compounds (Table 1) and literature data. 

**Figure 3:**
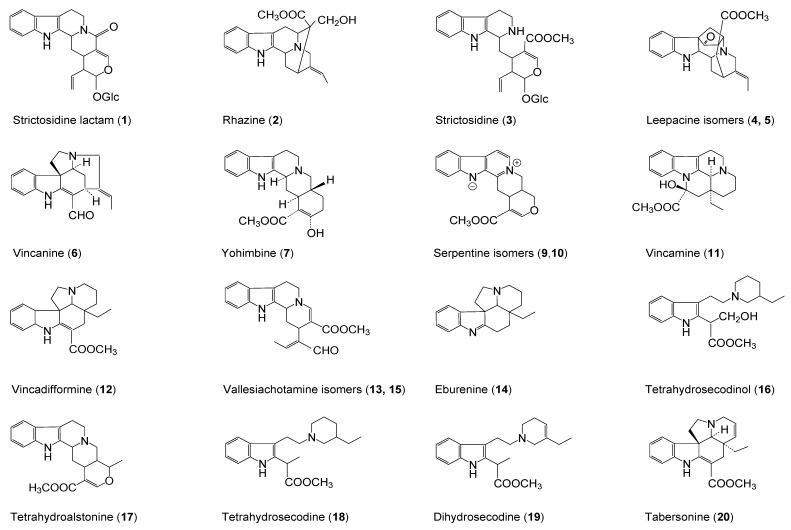
Structures of alkaloids identified by UPLC-PDA-MS. Numbers refer to compounds in Table 2. Compounds **6**, **7,** **11** and **20** were identified by direct comparison with authentic reference samples, thus their structures are depicted with assignment of relative configurations. All other compounds, including an isomer (**8**) of yohimbine (**7**), were tentatively assigned based on their mass spectra and are therefore depicted without stereochemical assignment.

Compound **1** (Table 2, Figure 3, Figures S1 and S2) was identified as strictosidine lactam. Compound **2** was identified as rhazine on the basis of UV and MS spectra. Compound **3,** which had a UV spectrum closely similar to that of alkaloid **1** (Figure S1), exhibited a protonated molecular ion [M + H]^+^ at *m*/*z* 531 in positive ion mode. Occurrence of the characteristic fragment ion *m*/*z* 514 ([M + H − OH]^+^) indicated the cleavage of a hydroxyl group (Figure S2). In negative ion mode, the corresponding deprotonated ion [M − H]^−^ was detected at *m*/*z* 529. This alkaloid was identified as strictosidine (Table 2, Figure 3).

Based on UV and MS data, compounds **6** and **14** were identified as vincanine and eburenine (Table 1 and Table 2, Figure 2, Figures S1 and S2). Comparison of the ESI^+^ signals of vincanine (**6**; channel *m*/*z* 293, Figure 2) and eburenine (**14**; channel *m*/*z* 281) showed equally high intensities. However, the UV chromatogram and total scan iongrams exhibited considerable peak tailing (Figure 1A–C). Thus, the neighbouring sharp peak on the right side of vincanine was strongly distorted at the UV maximum (365 nm) and at [M + H]^+^ *m*/*z* 293 of vincanine (Figure 1B). The [M + H]^+^ ion of eburenine at *m*/*z* 281 similarly interfered with the separation of vallesiachotamine isomers (Figure 1B, Table 2).

Compound **7** had the same retention time in the UPLC-MS analysis of *Rhazya* extract (Table 2) as the pure yohimbine reference substance (Table 1). In addition to compound **7**, another yohimbine isomer (**8**) was detected having similar UV and MS spectra (Table 2, Figures S1 and S2). The intensity of these isomers displayed at channel *m*/*z* 355 was among the lowest of all alkaloids (Figure 2). A further yohimbine-like compound with an [M + H]^+^ ion at *m*/*z* 355 eluted at 3.26 min, but the MS spectrum was considerably distorted by extra ions at *m*/*z* 297 (100) and 293 (38).

Compounds **9** and **10** (Figure 3) had identical UV maxima (Figure S1) and molecular ions at *m*/*z* 349 (ESI^+^) (Figure S2) and *m*/*z* 347 (ESI^−^) (Table 2). The compounds were identified as serpentine isomers. Isomer I had a higher intensity than isomer II (Figure 2).

In the UV and total ion chromatogram (Figure 1A,B), the vincamine peak (**11**, Table 2) in *Rhazya* extracts was hardly visible due to co-elution with the adjacent compound. However, the extracted ion channel at *m*/*z* 355 (Figure 2), showed the same retention time (t_R_) at 5.03 min as the reference compound (Table 1). Despite its very low intensity, vincamine displayed a rather symmetric peak with baseline separation.

Compounds **13** and **15** exhibited equal UV maxima and had a base peak ion [M + H − CH_3_OH]^+^ at *m*/*z* 319 in positive ion mode (Table 2). Based on UV and MS data, these compounds were identified as vallesiachotamine isomers. Extracted ion recording (ESI^+^ channel *m*/*z* 351; Figure 2) also showed a peak zone at 1.9–2.1 minutes composed of two alkaloids corresponding to leepacine isomers (**4**, **5**). Both UV and mass spectra are presented in Figures S1 and S2. On the basis of UV and MS spectra, compound **17** was identified as tetrahydroalstonine.

Compounds **16**, **18** and **19** constituted a specific group of alkaloids having a base peak fragment at *m*/*z* 126 (Table 2). No fragment ions at higher mass range were found after the molecular ions (Figure S2). Compound **16** displayed low-intensity UV maxima (Figure S1) and a protonated molecular ion [M + H]^+^ at *m*/*z* 359 in positive ion mode. The fragment ion at *m*/*z* 341 occurred due to the loss of water [M + H − H_2_O]^+^. This alkaloid was identified as tetrahydrosecodinol and constitutes a new record for *R. stricta*. Compound **18** had an identical UV absorbance with alkaloid **16** and showed typical fragmentation for tetrahydrosecodine (Table 2). Compound **19**, displaying an UV maximum at a higher wavelength of 305 nm and an [M + H]^+^ ion at *m*/*z* 341, was identified as dihydrosecodine. The structures of these compounds are illustrated in Figure 3. Some evidence for the presence of dimeric secamine-type alkaloids was also obtained. For example, the molecular ion *m*/*z* 681 with a typical base peak at *m*/*z* 126 and the parent monomer ion at *m*/*z* 341 was found at various retention times.

Compound **20** was identified as tabersonine based on comparison of UV and mass spectra and retention time with the authentic reference standard (Table 1 and Table 2). The structure of tabersonine is shown in Figure 3. In addition, another compound displaying similar UV spectrum as tabersonine was detected. By using extracted ion recording (channel *m*/*z* 339) from TIC, clone number 3 showed an abundant and symmetric peak at 7.1 min (Figure S3A,B) exhibiting UV absorption with three intense maxima and fragment ion *m*/*z* 307 due to the loss of methanol. No further fragments higher than the molecular ion (*m*/*z* 339) were detected (Figure S3C,D). The data suggest that this alkaloid is vincadifformine (**12**).

**Table 2:** Identification of indole alkaloids of *Rhazya stricta* hairy roots by UPLC-PDA-MS.

No	Compound	t_R_ (min)	UV (nm) λ_max_	MW	ESI^+^ (*m*/*z*) (rel. int. %)	ESI^−^ (*m*/*z*) (rel. int. %)	References
1	Strictosidine lactam	1.31	217 (sh), 227, 283, 290	498	337 (100), 499 (98), 267 (12)	497 (100), 335 (tr), 557(71)	[9,11,12,13,14]
2	Rhazine	1.77	227, 279, 291 (sh)	352	353 (100), 323 (14), 307 (10), 230 (5)	351 (100), 411 (15) ^a^, 319 (12)	[15,16]
3	Strictosidine	1.87	228, 270, 280, 290	530	531 (100), 514 (37), 369 (4)	529 (20), 589 (100) ^a^	[11]
4	Leepacine isomer I	1.95	208, 252, 305	350	351 (100)	349 (tr)	[1,9]
5	Leepacine isomer II	2.14	206, 250, 305	350	351 (100)	349 (tr)	[1,9]
6	Vincanine	2.46	246, 300, 365	292	293 (100)	291 (100)	[17,18]
7	Yohimbine	3.33	227, 279	354	355 (100), 212 (6), 144 (11), 224 (4)	353 (100), 413 (88) ^a^	[7,19]
8	Yohimbine isomer	3.63	226, 279	354	355 (100), 212 (11), 144 (21), 224 (2)	353 (100), 413 (34) ^a^	[7,19]
9	Serpentine isomer I	4.28	210, 248, 307, 368	348	349 (100)	347 (tr)	[9,20,21,22]
10	Serpentine isomer II	4.60	210, 248, 307, 368	348	349 (100)	347 (tr)	[9,20,21,22]
11	Vincamine	5.03	228, 280	354	355 (100), 337 (82)	n.d	[18,23]
12	Vincadifformione	7.01	227, 297, 328	338	339 (100), 307 (43)	n.d.	[24]
13	Vallesiachotamine isomer I	8.47	222, 292	350	319 (100), 281 (83) ^c^, 351 (50), 170 (35), 373 (18) ^b^	349 (100)	[25,26,27,28,29]
14	Eburenine	8.96	222, 262	280	281 (100)	n.d	[30,31]
15	Vallesiachotamine isomer II	8.99	223, 291	350	319 (100), 351 (56), 170 (49), 281 (30) ^c^, 373 (20) ^b^	349 (100)	[25,26,27,28,29]
16	Tetrahydrosecodinol	9.27	222, 283, 290	358	126 (100), 359 (62), 341 (49), 246 (30)	357 (tr)	[32]
17	Tetrahydroalstonine	14.45	227, 270 (sh), 282, 290	352	353 (100), 144 (60)	n.d	[30,33]
18	Tetrahydrosecodine	15.34	224, 283, 290 (sh)	342	126 (100), 343 (90), 230 (53)	n.d	[32]
19	Dihydrosecodine	15.99	224, 280 (sh), 290, 305	340	126 (100), 341 (51), 228 (26)	n.d	[32]
20	Tabersonine	19.25	229, 298, 331	336	337 (100), 305 (68), 228 (2)	n.d	[21,22,24]

^a^ acetate adduct; ^b^ sodium adduct; ^c^ interfered by eburenine; n.d: not detected; tr: trace.

## 3. Discussion

Generally, all alkaloids occur in multicomponent mixtures and the separation of alkaloids from other groups of natural products is the first requirement for comprehensive and detailed qualitative and quantitative analyses of single alkaloids. Acidic inorganic extraction solvents are commonly used, since they are known to improve the stability and solubility of alkaloids [34]. Therefore, this type of alkaloid-specific extraction method utilizing diluted sulphuric acid was applied in the study. Further sample preparation included alkalinization by diluted ammonia and subsequent extraction with organic solvent.

The development of LC conditions includes a large number of different parameters. For example, the selection of an appropriate solvent system and proportions of organic solvent and buffer solutions, concentration of buffer salt and ion pair reagent, and acidic or alkaline pH must be taken into account [4]. 

The solvent system of the UPLC method was tested under different conditions without ion pair reagent to obtain well-resolved peaks of major alkaloids. A good peak shape and sensitivity of various alkaloids was obtained from the repeated injections using the gradient condition (A: 10 mM ammonium acetate, pH 10; B: acetonitrile). This condition showing improved separation combined with alkaline pH and the use of a BEH C18 column agree well with the analysis of yohimbe bark alkaloids by [7], who reported that this column provided better separation compared to other tested columns. In addition, similar column performance has been reported for the UPLC analyses of alkaloids from *Lindera aggregata* [5], *C. roseus* [35] and *Coptidis* spp. [36].

In our previous study, transgenic hairy roots of *R. stricta* were developed for the investigation of their capacity to accumulate alkaloids. The alkaloid profile and contents of five major alkaloids in 20 transformed *R. stricta* hairy root clones were compared to non-transformed roots by data obtained from HPLC analysis [9]. HPLC analysis was performed according to the method described in [3] with a slightly modified gradient program. The use of phosphate buffer and heptanesulphonic acid as an ion exchange reagent resulted in a clearly improved separation of alkaloids. However, the use of phosphate buffer and heptanesulphonic acid is not compatible with LC-MS analysis, which was applied for the identification of the alkaloids. The identification of five major alkaloids in the *Rhazya* extract was therefore based on the resemblance of their UV data from HPLC analyses and UV and MS data from UPLC-MS.

In the present study, the focus was on comprehensive alkaloid profiling of *R. stricta* hairy roots, from three representative clones, by UPLC-MS. The method resulted in the identification of 20 alkaloids (Table 2). The identification of six alkaloids, vincanine, leepacine isomers, strictosidine lactam and serpentine isomers in our previous work [9] is further supported by their MS spectra (Figure S2). Strictosidine (**3**), vincanine (**6**) and eburenine (**14**) were the most abundant alkaloids (Figure 3). The alkaloids belong to nine subgroups including aspidosperma- (**12**, **14**, **20**), ajmaline- (**4**, **5**), eburnamine- (**11**), heteroyohimbine- (**9**, **10**, **17**), hunterburine-type (**13**, **15**), strictosidine- (**1**, **3**), sarpagine- (**2**), secodine- (**16**, **18**, **19**), strychnos- (**6**), and yohimbinoid- (**7**, **8**) type alkaloids [1,37]. Strictosidine, a monoterpene indole alkaloid glycoside, is a universal precursor of the terpenoid indole and related alkaloids and was first isolated from *R. stricta* [38]. Secodine-type alkaloids, derived in a series of reactions from strictosidine, are of considerable biogenetic interest as late stage intermediates in eburnamine-, aspidosperma- and strychnos-type alkaloid biosynthesis. The alkaloids occur in various stages of cyclisation, and in monomeric and dimeric forms [39,40]. 

Our investigation on alkaloid composition in *R. stricta* hairy roots, analysed by the LC-MS method reported here and the previously applied GC-MS method [10], has hitherto resulted in the identification of 31 terpenoid indole alkaloids, ten of which were detected exclusively with UPLC-MS. A list of the alkaloids, their identification method and the subgroup they belong to is presented in Table S1.

Higher selectivity and sensitivity of MS can be achieved through extracting ions from total ion chromatograms (TIC) [41]. This is useful for revising data to detect isomers, resolve co-eluting substances and detect minor compounds. In the current study, extracted ion recording was applied to detect minor alkaloids, e.g., alkaloids **4**, **5**, **7**–**10**, alkaloids exhibiting the same molecular ion or peaks (e.g., **7**, **8**, **11**) or those eluting closely*,* e.g., **13**, **14** (Figure 2). Extracted ions from TIC provide clean chromatograms and sharp peaks of compounds of interest; therefore, TIC is widely used for quantification of compounds, too, e.g., alkaloids in *C. roseus* [20,21].

An HPLC method had been applied for the separation of crude extracts of *Rhazya* cell cultures [24] and strictosidine lactam was among the major alkaloids found. In this study, both UV and LC-MS spectra of this alkaloid were consistent with those reported in a number of papers investigating other plant species such as *Ophiorrhiza pumila* [11], *Nauclea pobeguinii* [12] and *Nauclea latifolia* [13] or the metabolism of strictosamide in animal studies [14]. Strictosidine lactam (**1**) is another major alkaloid in *Catharanthus roseus* [42], and it has also been identified in the hairy root cultures of *C. roseus* along with tabersonine, tetrahydroalstonine and yohimbine [43]. Strictosidine (**3**), which has been long known as an intermediate in the biosynthesis of indole alkaloids [38,39], was among the principal alkaloids in *Rhazya* extract. Its UV and MS data were in full accordance with those reported earlier [11]. 

Rhazine (syn. akuammidine) was among the first alkaloids to be isolated from *R. stricta* [44]. In the current study, the UV and MS data of rhazine (**2**) were in line with those obtained by ion trap time-of-flight mass spectrometric (LC-MS-IT-TOF) analyses of *Alstonia scholaris* [15,16].

Leepacine isomers (**4**, **5**) showed identical UV spectra and also the molecular ion *m*/*z* 351. These alkaloids can be distinguished from vallesiachotamine on the basis of their retention time and UV absorbance. The two main UV maxima corresponded to literature values [1,45] and the third one was shifted slightly towards higher wavelength as indicated in Figure S1. It is known that UV maxima of *Rhazya* alkaloids can shift to a slightly higher wavelength in alkaline conditions [46]. At pH 6, leepacine has shown a third UV maximum at 298 nm [45]. The low-intensity UV maximum at 363 nm was probably caused by the interference of vincanine (**6**). 

Vincanine (**6**) was present as a main component together with eburenine (**14**). The UV and MS data (Table 2) were in accordance with those obtained from pure substance and HR-MS analyses of isolated vincanine from *R. stricta* [17] and from *Vinca* spp. [18]. Vincanine, also called (−)-nor-*C*-fluorocurarine, belongs to strychnos-type alkaloids, and was already isolated in the 1960s from *Diplorhynchus condylocarpon* [47]. Since then, it had been discovered in *R. stricta* [17] and later as a major alkaloid in its cell culture [24]. 

Yohimbine isomers have previously been isolated from suspension cultures of *R. serpentina* plants [48], whereas they have not been identified in *R. stricta*. Yohimbine isomers (compounds **7** and **8**) possess a tetrahydro-β-carboline moiety and have a pronounced deprotonated molecular ion *m*/*z* 353 in ESI^−^. In addition, the UV and ESI^+^ spectra (Table 2) were also fully consistent with those of the reference compound and reported from yohimbe bark [7,19]. Using the UPLC-MS method, two yohimbine isomers (**7**, **8**) with similar fragmentation patterns were separated with retention times very close to each other in our study.

Serpentine is a quaternary alkaloid, which is typical for *Rauwolfia* [49] and *Catharanthus* [50] species, but it has not been reported earlier in *Rhazya* species. In the current study, extracted ion recording in ESI^+^ and ESI^−^ revealed two peaks. Isomer I showed higher intensity than isomer II (**9**, **10**). The UV and ESI^+^-MS data (Table 2) were consistent with serpentine from *C. roseus* extracts analysed by ESI-IT-MS [20] and by HPLC-ESI-MS/MS [21,22]. 

Vincamine (**11**), a member of the eburnamine-type alkaloids, has not been reported in intact *Rhazya* plants or cell cultures. Vincamine showed the same retention time, fragmentation pattern, UV and ESI^+^-MS data as the pure reference compound. Furthermore, these data were also in line with those reported in the literature [18,23]. 

In the present study, two isomers of vallesiachotamine (**13**, **15**) were identified from *R. stricta* hairy root extracts. The mass spectra of the isomers exhibited typical fragments, [M + H]^+^ at *m*/*z* 351 and a fragment of tetrahydro-β-carboline moiety at *m*/*z* 170. These isomers possess a strong UV absorption in the 290 nm region, which is characteristic for hunterburine-type alkaloids [25]. Both UV and MS profiles of the isomers (Table 2) were in line with EI-MS or HR-MS data reported from *R. stricta* [26,27,28] and additionally with those of *Vallesia dichotoma* [25] and *Strychnos tricalysioides* [29]. Detailed isomeric analyses by NMR have shown that the hydrogen linked to C19 is in the *trans* position in vallesiachotamine but *cis* in isovallesiachotamine [29]. These isomers have also been identified in *R. stricta* cell suspension cultures [24,28,51,52]. 

Spectral data of tetrahydroalstonine (**17**) was consistent with data reported in the literature [30,33]. This compound has previously been isolated from *R. stricta* but has not been reported from *Rhazya* cell cultures.

The group of secodine alkaloids include tetrahydrosecodine (**18**) and dihydrosecodine (**19**) together with tetrahydrosecodinol (**16**), which was identified in *R. stricta* for the first time. The base peak fragment *m*/*z* 126 is typical for alkaloids containing a secodine skeleton with a saturated (**16**, **18**) or unsaturated (**19**) piperidine ring [32]. The molecular ion *m*/*z* 358 of tetrahydrosecodinol, obtained by EI-MS, readily loses water and produces a radical ion *m*/*z* 340 corresponding to [M^+^] of 15,20-dihydrosecodine [53]. This structure also allowed the double bond to be located in the piperidine unit. The present UV and MS data of tetrahydrosecodinol (**16**), tetrahydrosecodine (**18**) and dihydrosecodine (**19**) were consistent with the data from pure compounds isolated from *Rhazya* species [32].

Three indicative MS fragments including molecular ion, base peak (*m*/*z* 126) and parental ion for dimeric alkaloids were detected. The fact that these fragments were encountered at different retention times can be due to the incompatible pH of the eluent leading to several broadened peak zones spread along the baseline. Dimeric secamine and presecamine alkaloids with similar MS spectra have been reported in *Rhazya* already long ago [53,54]. Secodine is a presumed precursor of the dimeric group, which in turn produces the secamine group alkaloids [55]. Presecamines can be formed by the non-enzymatic dimerization of secodine units, either in the cell or during extraction [53,55].

Tabersonine (**20**), belonging to aspidosperma-type alkaloids, has been reported from *R. orientalis* leaves [56] and *R. stricta* cell cultures [24,52]. Vincadifformine (**12**), called earlier 6,7-dihydrotabersonine, is another member of this family [31]. Vincadifformine and eburenine (**14**) have been isolated from leaves of *R. stricta* and detected from the crude extract of its hybrid cell cultures by HPLC analysis [24,52]. In the current study, the spectral data of **20** was the same as in the pure reference compound and was in line with data reported in the literature [11,12,13,14,15,16,17,18,21,22,24,42,43,44,45,46,47,48,49,50]. The UV and MS data of **12** (Figure S3) was in accordance with those of *Rhazya* cell cultures [24].

## 4. Experimental Section

### 4.1. Chemicals

All solvents used for the extraction and chromatographic analysis were of analytical grade. Ammonium acetate, formic acid and sulphuric acid (95%–97%) were purchased from Sigma-Aldrich (St. Louis, MO, USA), ammonia solution 25% from Merck (Darmstadt, Germany) and dichloromethane (DCM) from Rathburn Chemicals (Walkerburn, UK). Vincanine was purchased from Accurate Chemical & Scientific Corporation (Westbury, NY, USA), vincamine, tabersonine, and yohimbine hydrochloride from Sigma-Aldrich. Purified water was obtained by PURELAB Ultra Analytic Water Purification System (ELGA LabWater, High Wycombe, UK).

### 4.2. Plant Material

Seeds of *R. stricta* were collected from Iran (Hormozgan province, Minab zone in the Persian Gulf area 27°08′48″ N, 57°04′48″ E). In order to obtain hairy roots, *R. stricta* leaves from seedlings were co-cultivated with wild type *A. rhizogenes* strain LBA 9402 as described in [9].

### 4.3. Extraction

Alkaloids were extracted from hairy roots as described in [9]. Briefly, alkaloids were extracted from the lyophilized powdered samples by adding 10% sulphuric acid (*v*/*v*). The acidic supernatants were basified to pH 10 with 25% ammonia solution. Alkaloids were then extracted with dichloromethane. The extracts were evaporated to dryness under nitrogen flow and dissolved into 500 µL methanol to be injected to UPLC-MS.

### 4.4. UPLC-PDA-MS

The UPLC-PDA-MS analyses were performed on a Waters Micromass Quattro micro™ triple quadrupole mass spectrometer with electrospray source combined with Waters Acquity Ultra Performance LC (UPLC) with photodiode array detector (PDA). The column was an Acquity UPLC™ BEH C18 (100 mm × 2.1 mm, 1.7 µm) with a precolumn and the analyses were performed at 25 °C. The solvent system was made alkaline as described earlier [57]. It consisted of a mixture of 10 mM ammonium acetate (pH 10; A) and acetonitrile (B). The flow rate was 0.4 mL/min. 

Two isocratic runs were tested using either 55% or 65% solvent A (10 mM ammonium acetate, pH 10 in water) and 45% or 35%, respectively, solvent B (acetonitrile). 

Three gradient runs were also tested. The solvent proportions were changed in 15 min from 35% to 50% (solvent B). In the first gradient run, the solvent system contained 0.1% formic acid in water (solvent A) and acetonitrile (solvent B). In the second gradient run, the concentration of formic acid in water was tenfold (1%). In the last gradient run, the solvents contained 10 mM ammonium acetate, pH 10 in water (solvent A) and acetonitrile (solvent B).

The analyses were performed both in positive (ESI^+^) and negative (ESI^−^) electrospray ionization mode and the data were collected in a mass range of *m*/*z* 100–850. The capillary and cone energies were 2.5 kV and 40 V in ESI^+^ and 2.5 kV and 20 V in ESI^−^ mode, respectively. Source temperature was 125 °C and desolvation temperature 350 °C. Desolvation gas flow was 800 L/h. The PDA detector was scanning wavelengths from 200 to 420 nm. 

## 5. Conclusions

In conclusion, an UPLC-MS method was developed for the determination of more polar terpenoid indole alkaloids (TIAs) in crude extracts of *R. stricta* hairy roots. A total of 20 TIAs belonging to nine subgroups were identified. Among them, tetrahydrosecodinol has not been previously reported from *R. stricta*. Strictosidine and secodine-type alkaloids, two main intermediates in the biosynthesis of TIAs, were detected too. Extracted ion recording was used to detect the compounds with the same molecular ion, minor and co-eluting components. The method presented here is applicable for the separation and identification of alkaloids in *R. stricta* hairy roots.

## Supplementary Materials

Supplementary materials can be accessed at: http://www.mdpi.com/1420-3049/20/12/19873/s1.
