# Supplementary material for: Analysis of Indole Alkaloids from Rhazya stricta Hairy Roots by Ultra-Performance Liquid Chromatography-Mass Spectrometry
Source: Molecules. 2015 Dec 17;20(12):22621–34. doi: 10.3390/molecules201219873 (PMC6332301; doi:10.3390/molecules201219873)
Supplement: Supplementary file 1 [file molecules-20-19873-s001.pdf]

Supplementary Materials: Analysis of Indole Alkaloids from *Rhazya stricta* Hairy Roots by Ultra-Performance Liquid Chromatography-Mass Spectrometry

Amir Akhgari, Into Laakso, Tuulikki Seppänen-Laakso, Teijo Yrjönen, Heikki Vuorela, Kirsi-Marja Oksman-Caldentey and Heiko Rischer

Table S1. Alkaloids detected in *R. stricta* hairy root extracts by LC-MS or GC-MS.

No	Alkaloids	Identified with UPLC-MS	Identified with GC-MS	Alkaloid Type
1	Strictosidine lactam	+	-	strictosidine
2	Rhazine *	+	+	sarpagine
3	Strictosidine	+	-	strictosidine
4	Leepacine isomer I	+	-	ajmaline
5	Leepacine isomer II	+	-	ajmaline
6	Vincanine *	+	+	strychnos
7	Yohimbine *	+	+	yohimbinoid
8	Yohimbine isomer *	+	+	yohimbinoid
9	Serpentine isomer I	+	-	heteroyohimbine
10	Serpentine isomer II	+	-	heteroyohimbine
11	Vincamine *	+	+	eburnamine
12	Vincadifformione *	+	+	aspidosperma
13	Vallesiachotamine isomer I *	+	+	hunterburine
14	Eburenine *	+	+	aspidosperma
15	Vallesiachotamine isomer II *	+	+	hunterburine
16	Tetrahydrosecodinol	+	-	secodine
17	Tetrahydroalstonine *	+	-	heteroyohimbine
18	Tetrahydrosecodine	+	-	secodine
19	Dihydrosecodine	+	-	secodine
20	Tabersonine	+	-	aspidosperma
21	Decarbomethoxytabersonine	-	+	aspidosperma
22	Aspidospermidine	-	+	aspidosperma
23	Eburnamenine	-	+	eburnamine
24	Quebrachamine	-	+	aspidosperma
25	Tubotaiwine	-	+	aspidospermatin
26	Condylocarpine	-	+	aspidospermatin
27	Dihydrovincadifformine	-	+	aspidosperma
28	Vincadine	-	+	aspidosperma
29	Pleiocarpamine	-	+	pleiocarpaman
30	Fluorocarpamine	-	+	pleiocarpaman
31	Ajmalicine	-	+	heteroyohimbine

+: Alkaloid was detected with the method. -: alkaloid was not detected with the method. *: Alkaloid which was detected by both LC-MS and GC-MS methods.

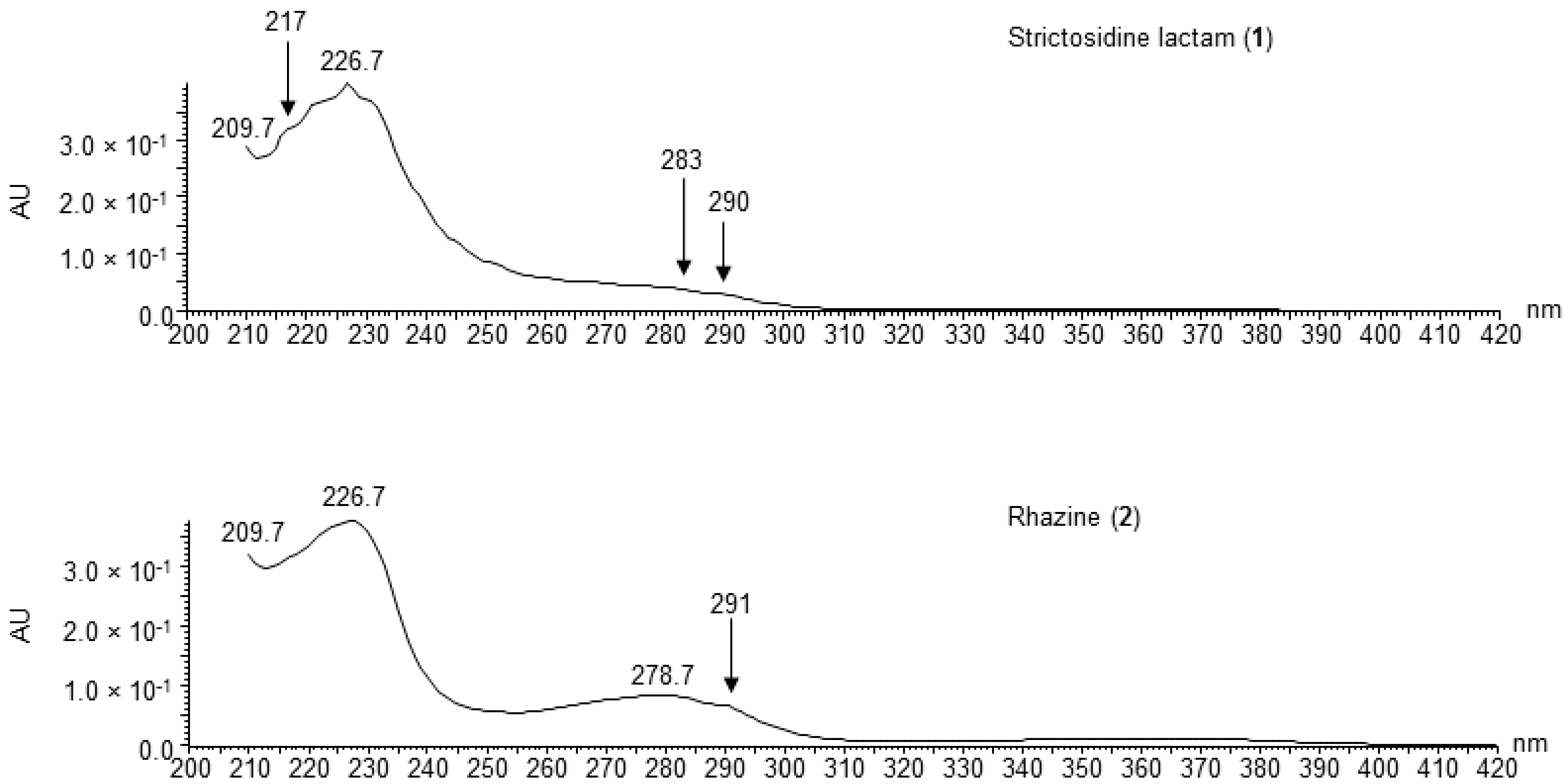

Figure S1. *Cont.*

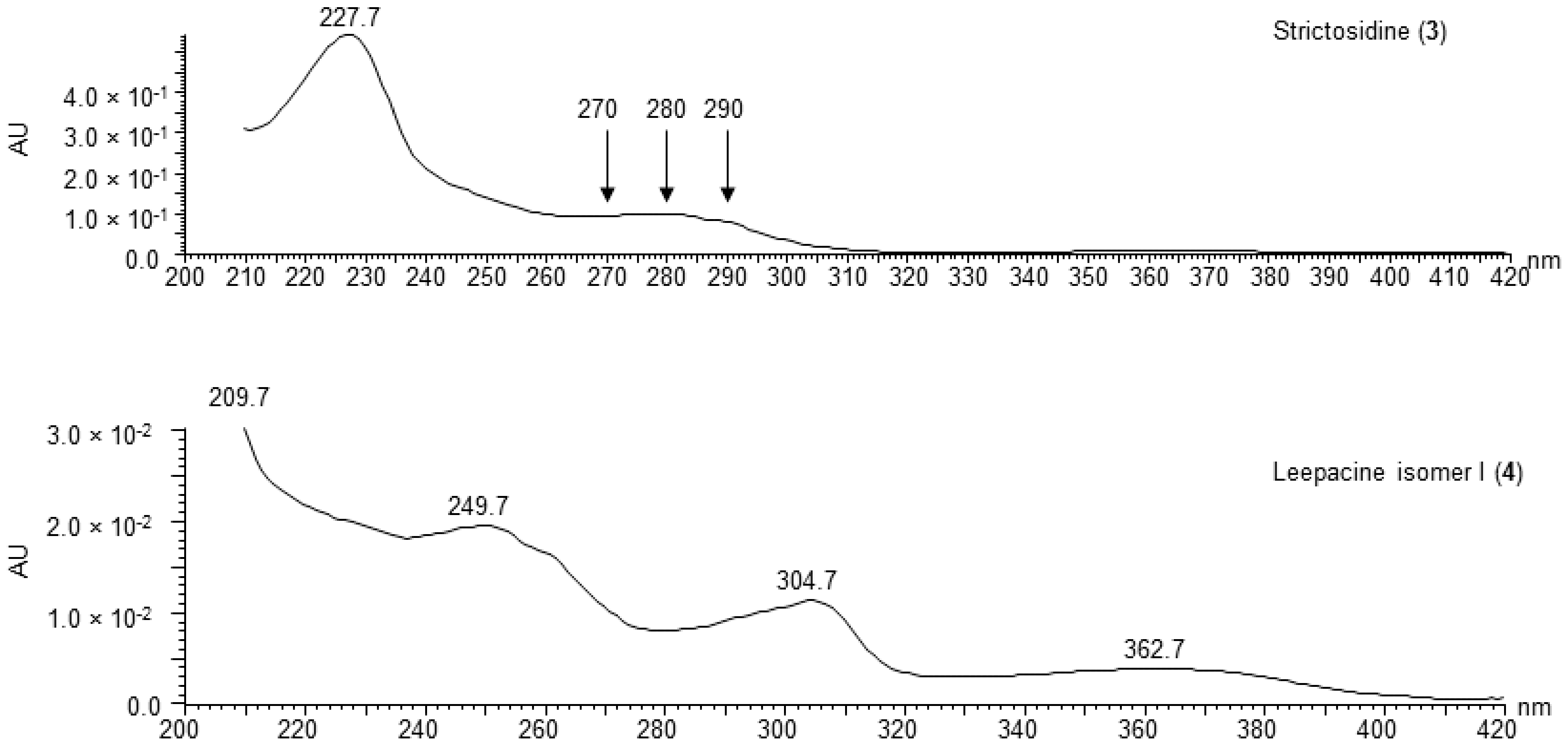

Figure S1. *Cont.*

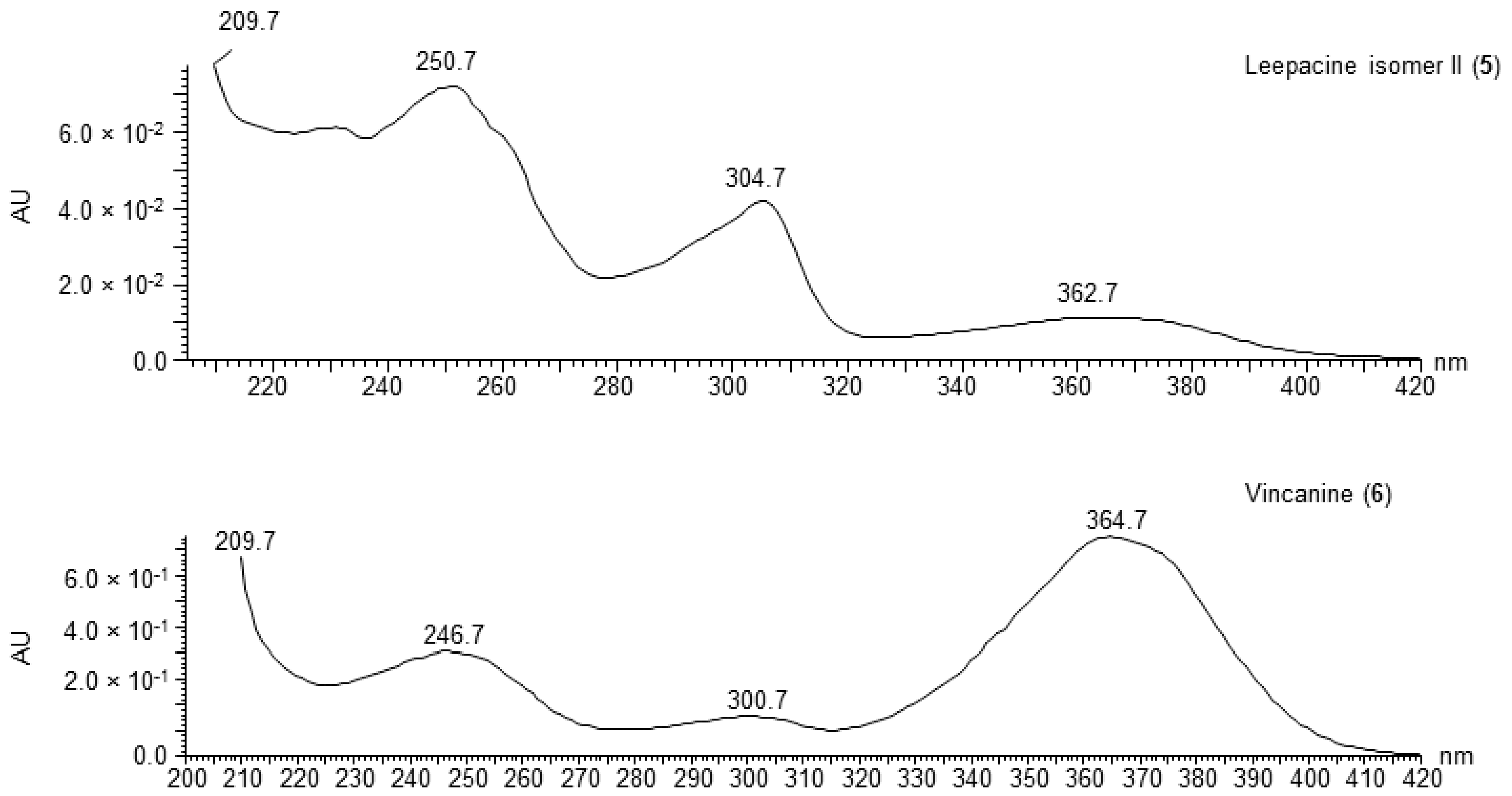

Figure S1. *Cont.*

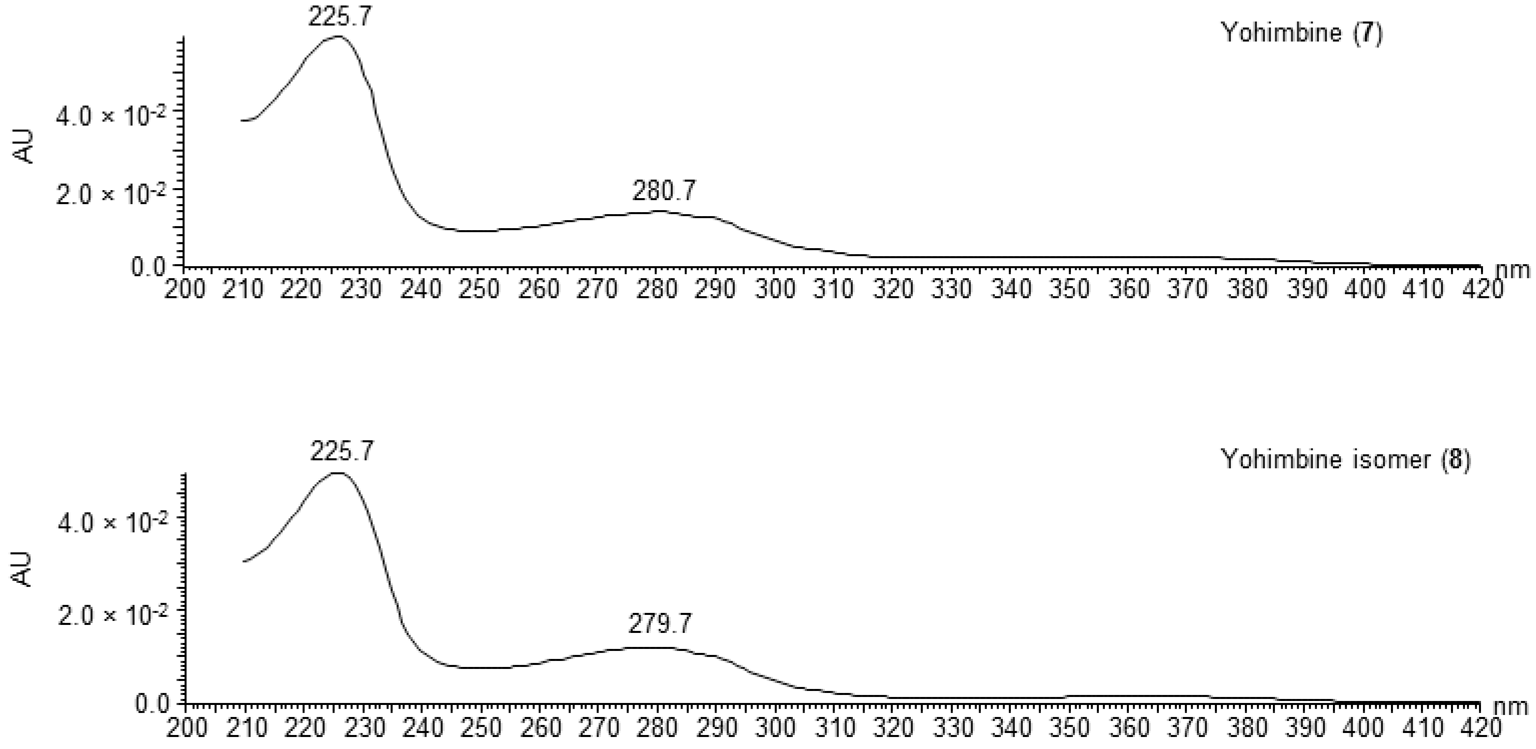

Figure S1. *Cont.*

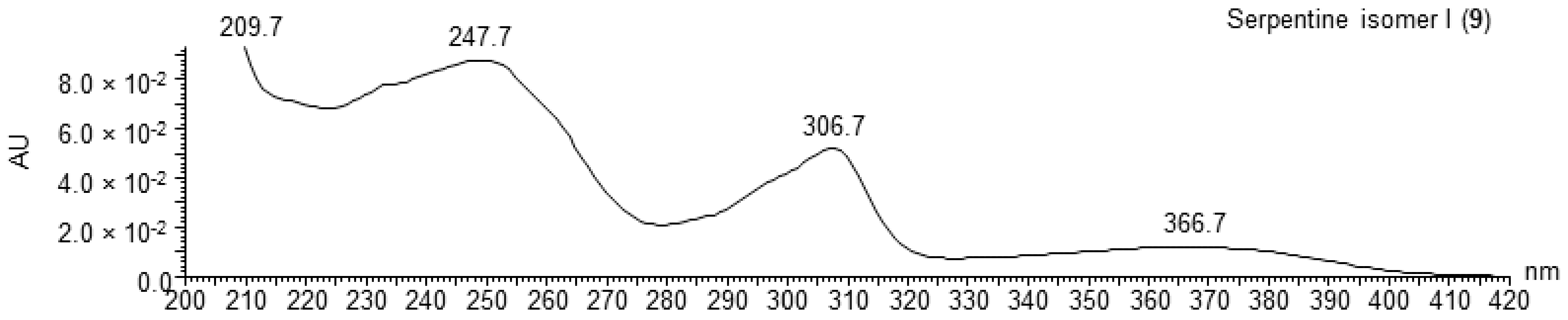

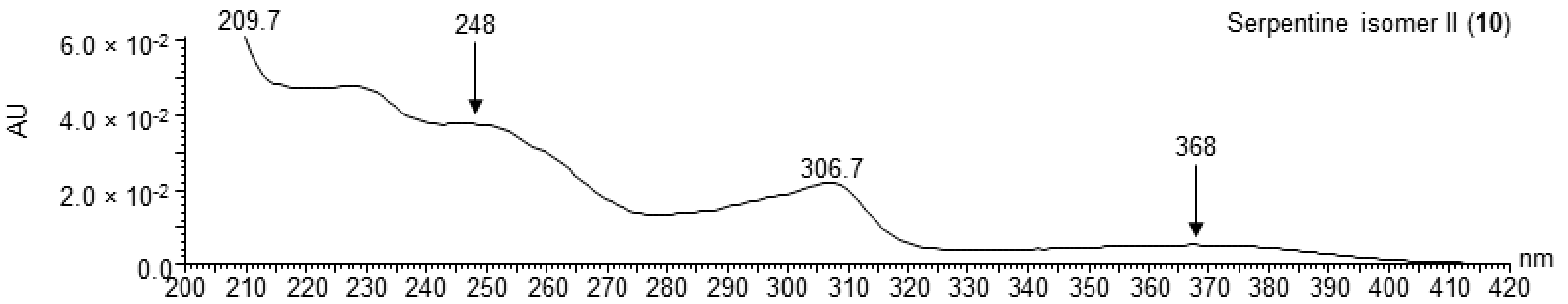

Figure S1. *Cont.*

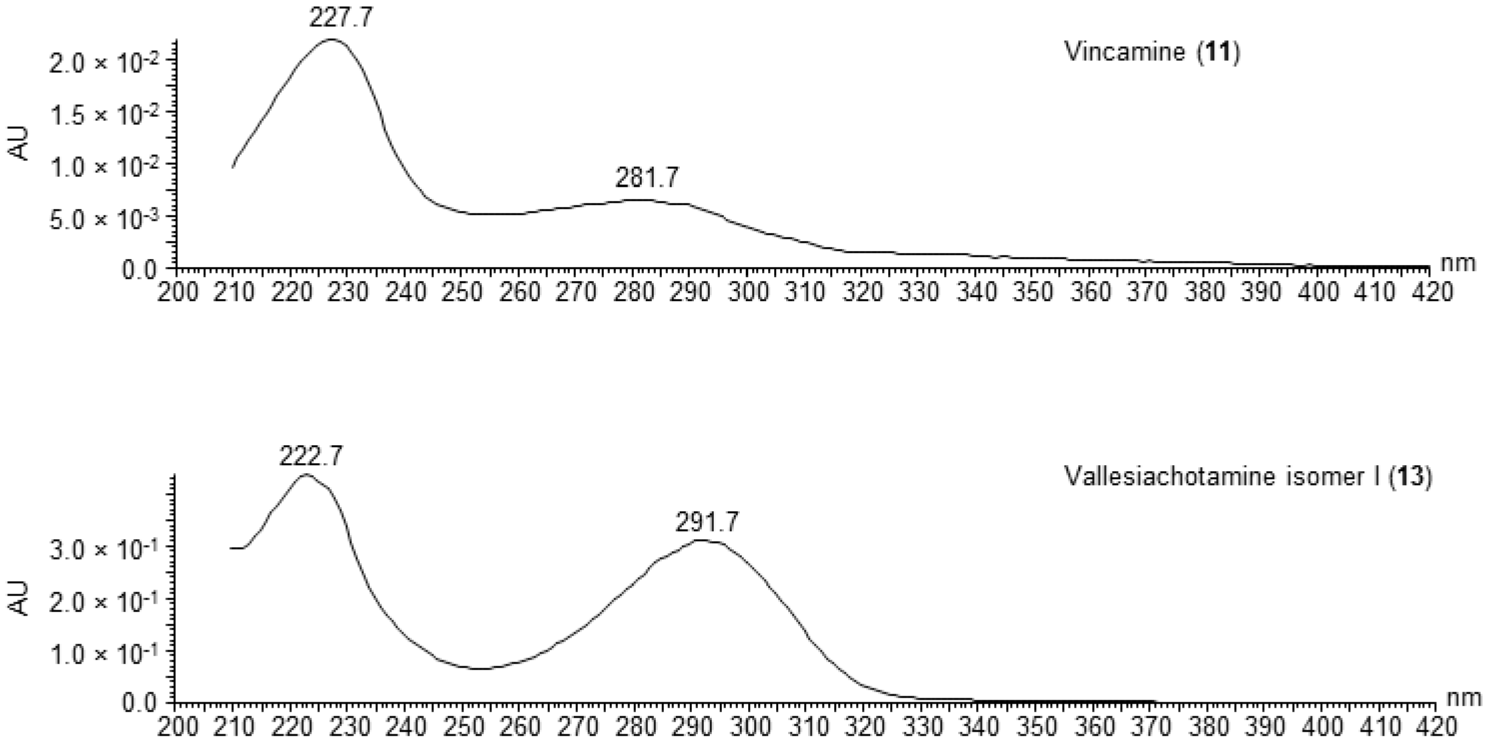

Figure S1. *Cont.*

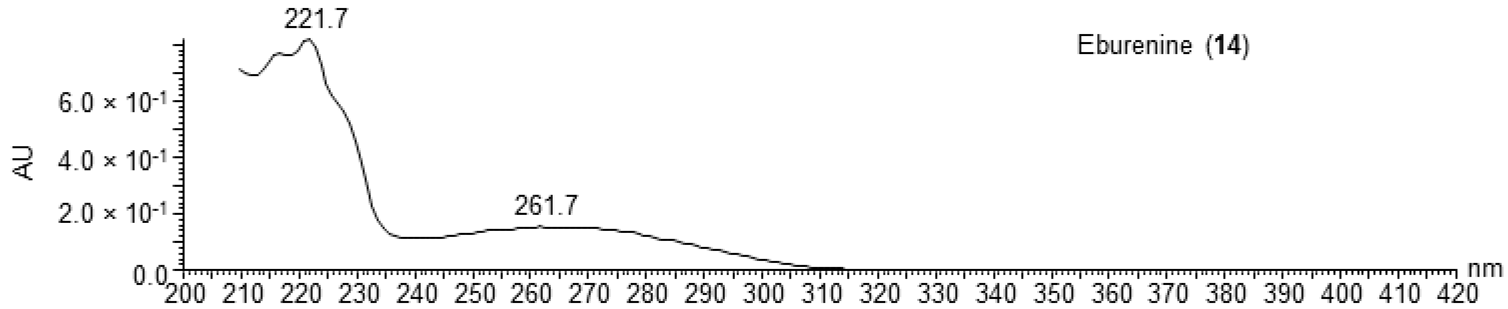

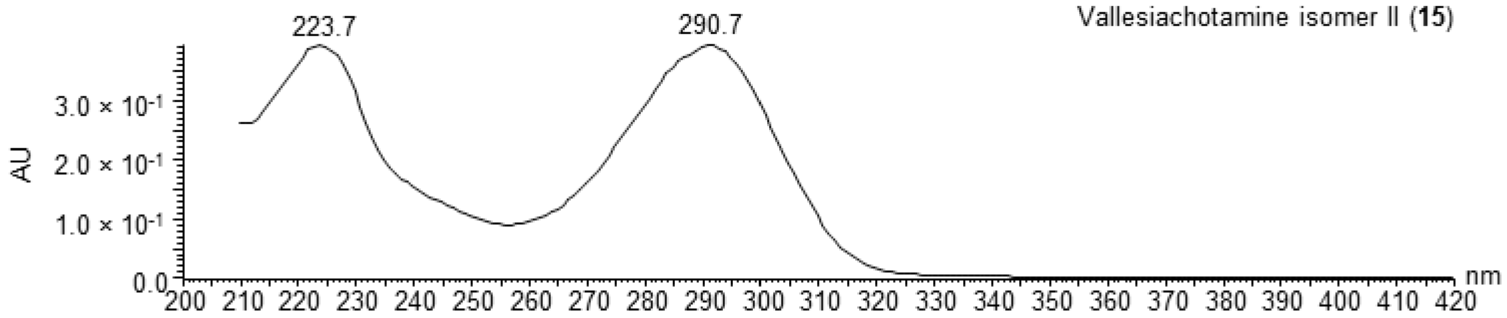

Figure S1. *Cont.*

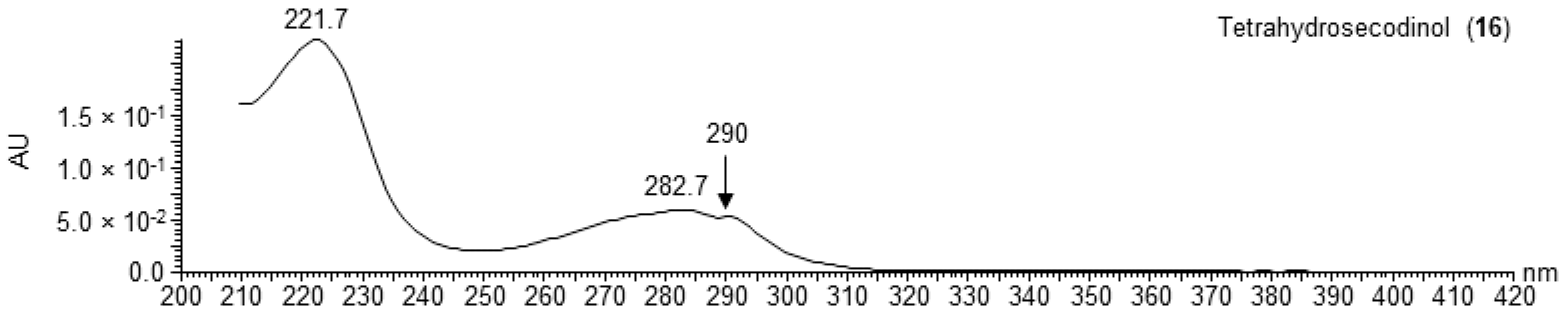

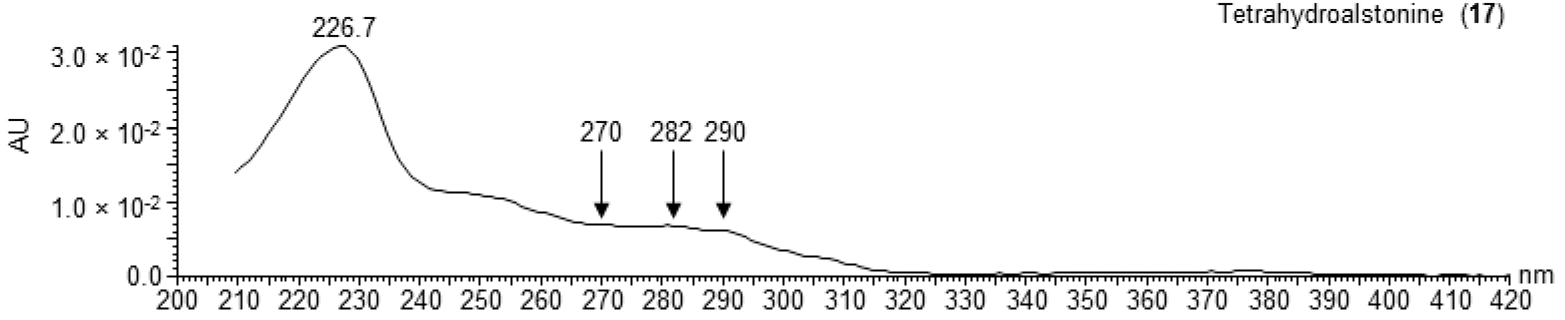

Figure S1. *Cont.*

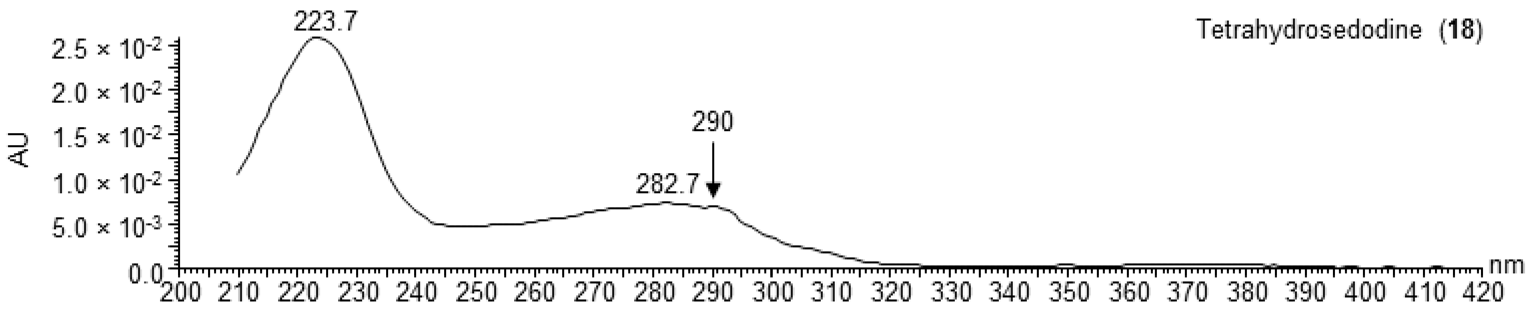

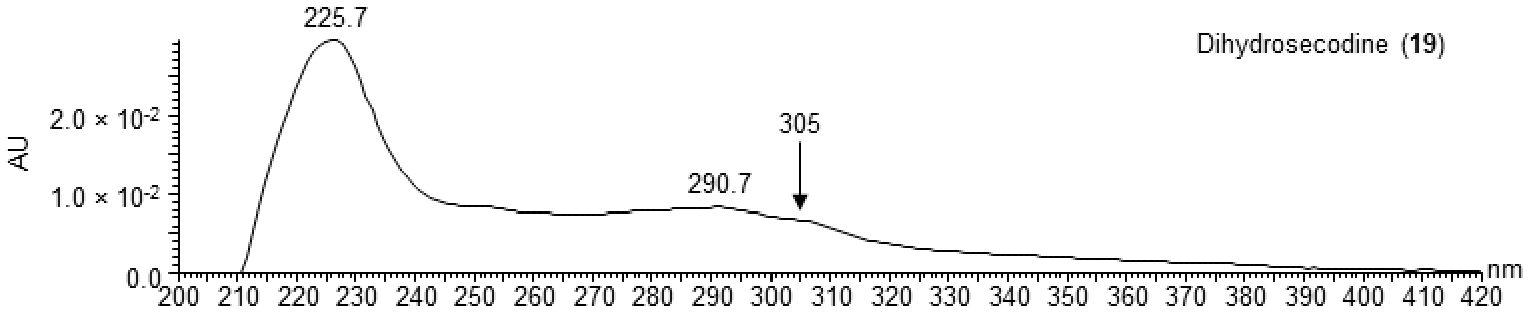

Figure S1. *Cont.*

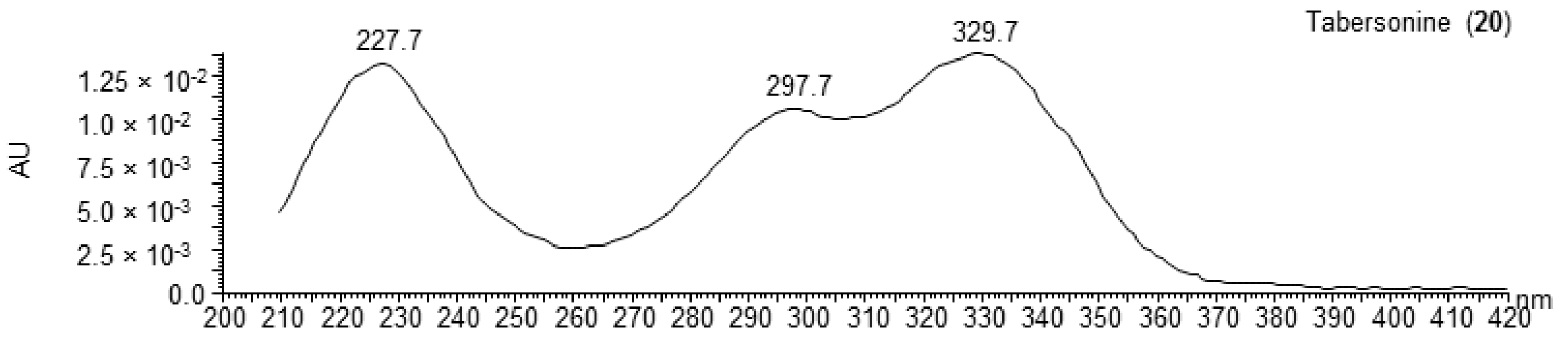

Figure S1. UV spectra of *R. stricta* alkaloids in UPLC-UV analysis. Data for vincadifformine (**12**) is presented in Figure S3.

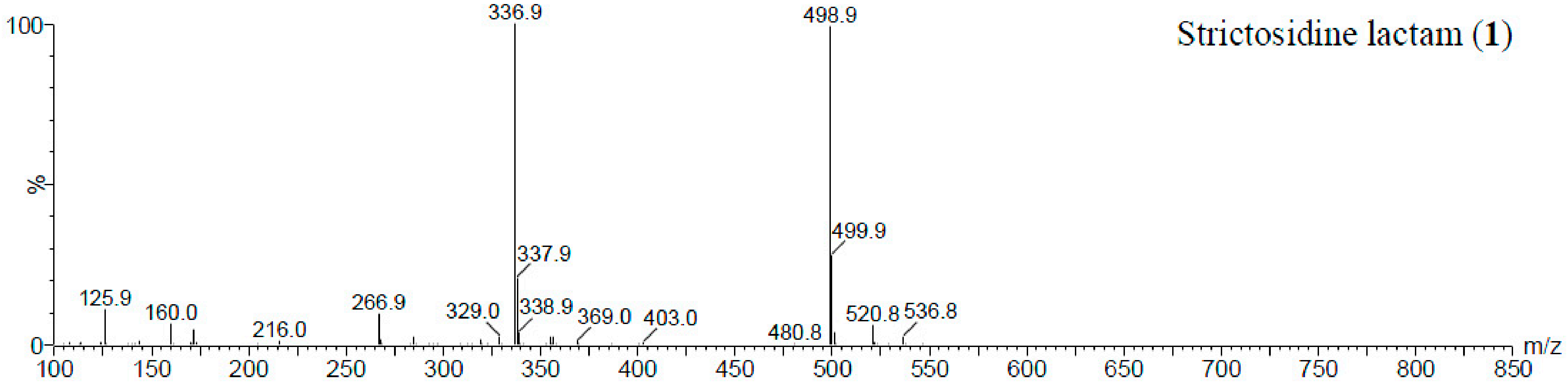

Figure S2. *Cont.*

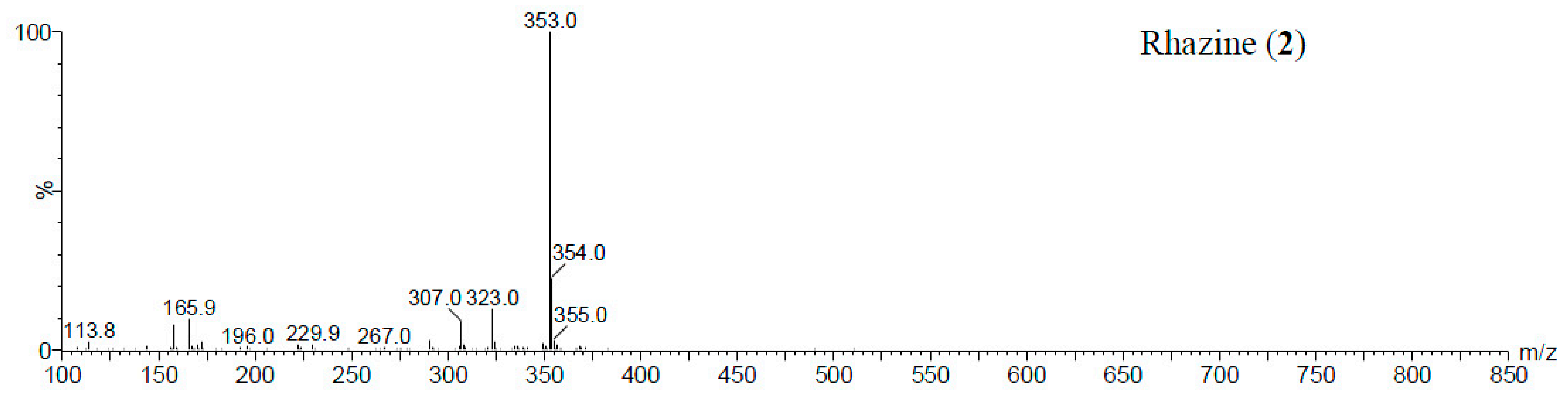

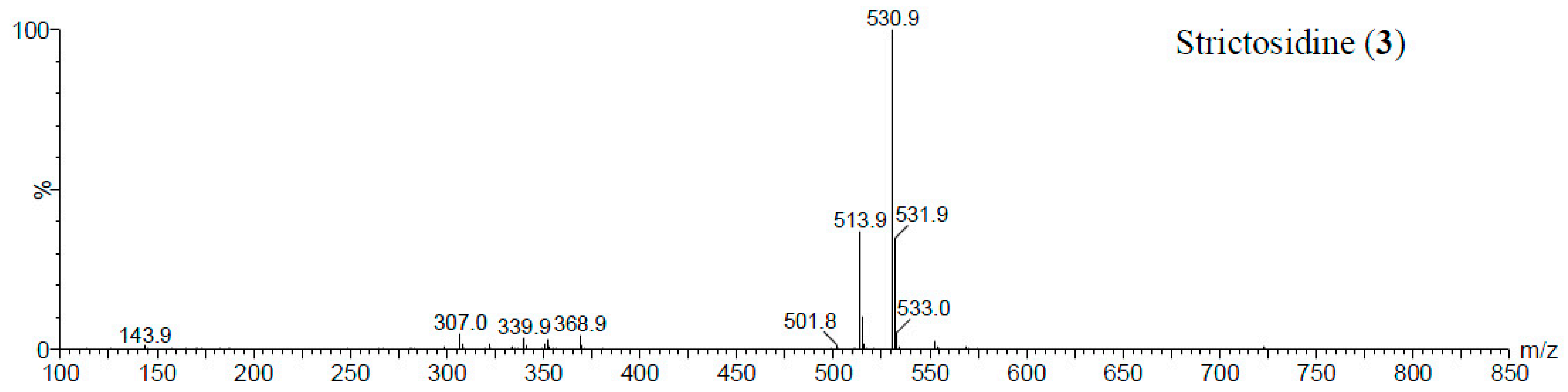

Figure S2. *Cont.*

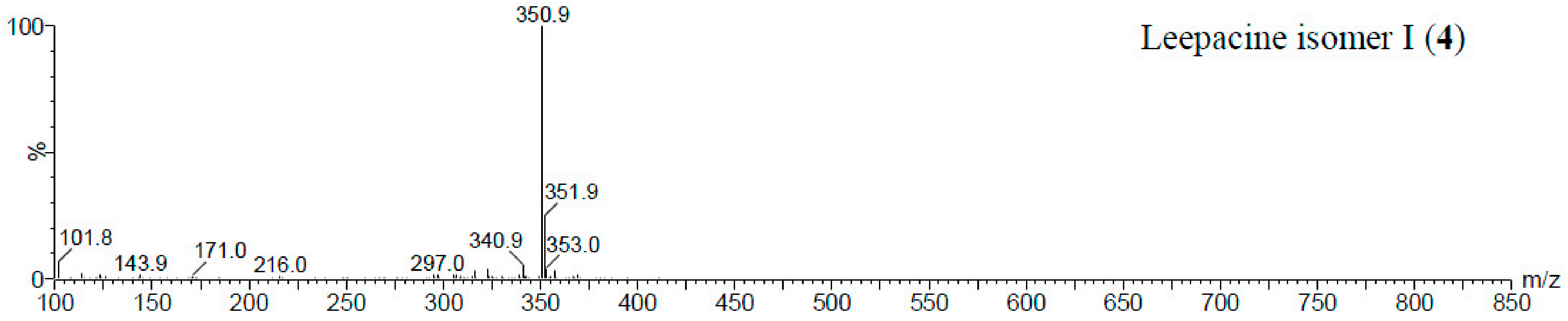

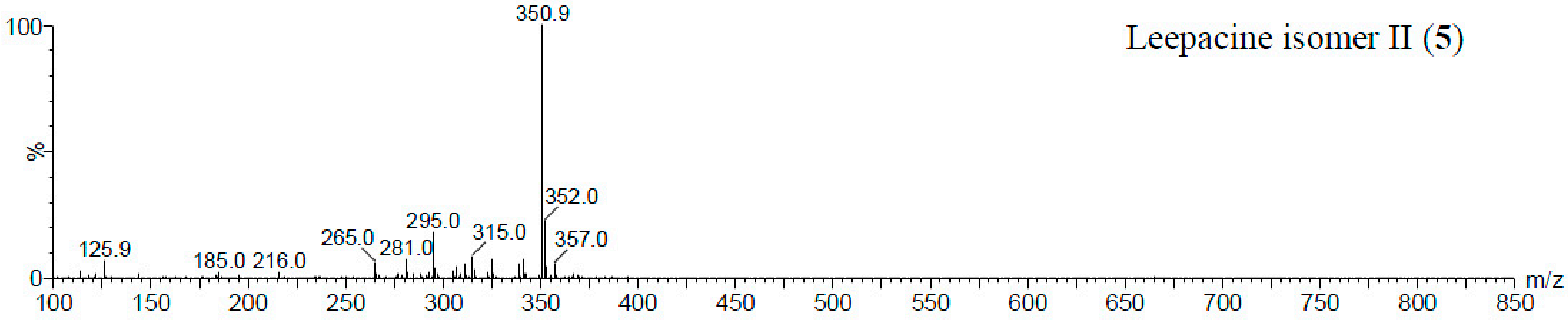

Figure S2. *Cont.*

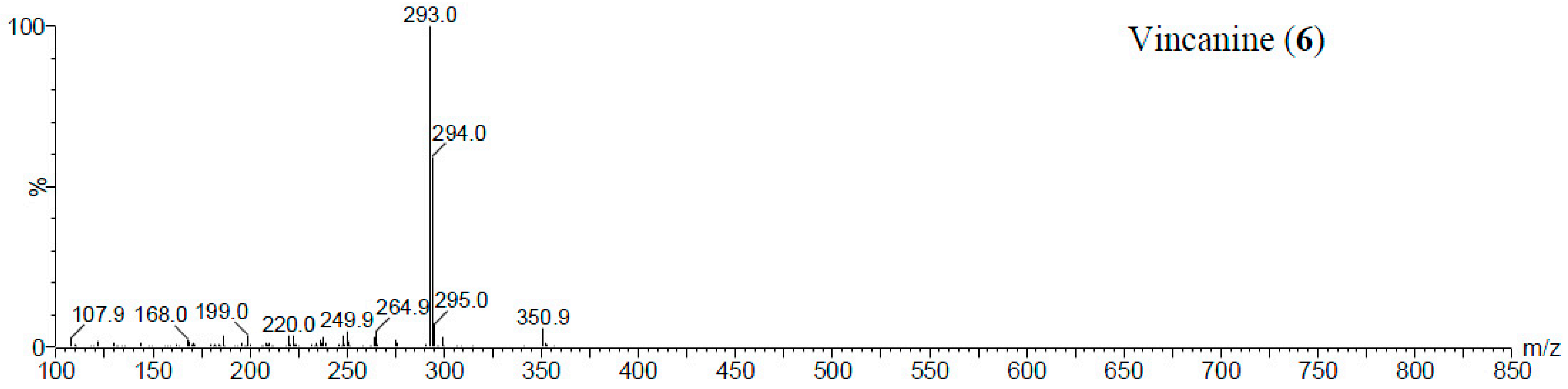

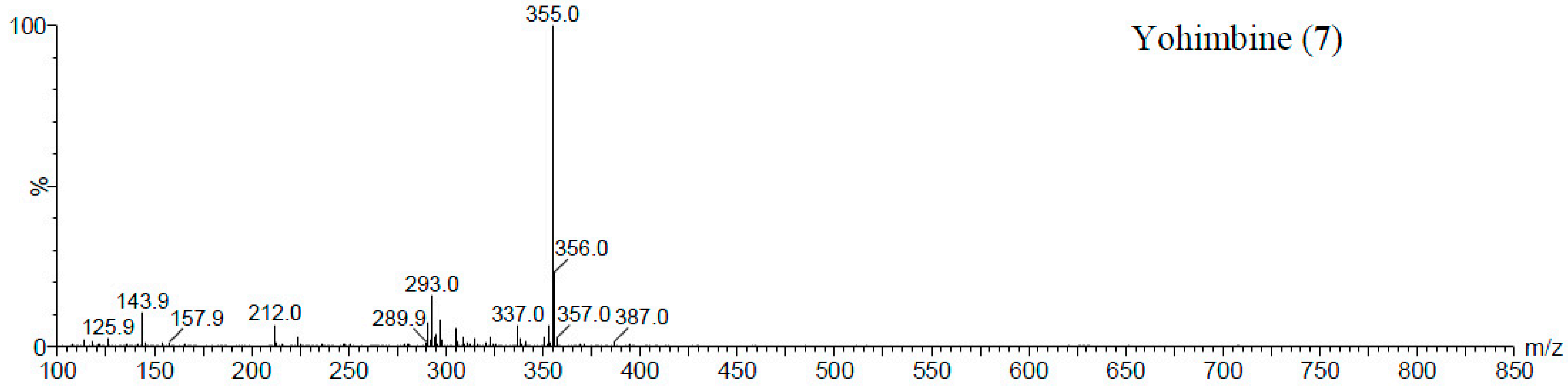

Figure S2. *Cont.*

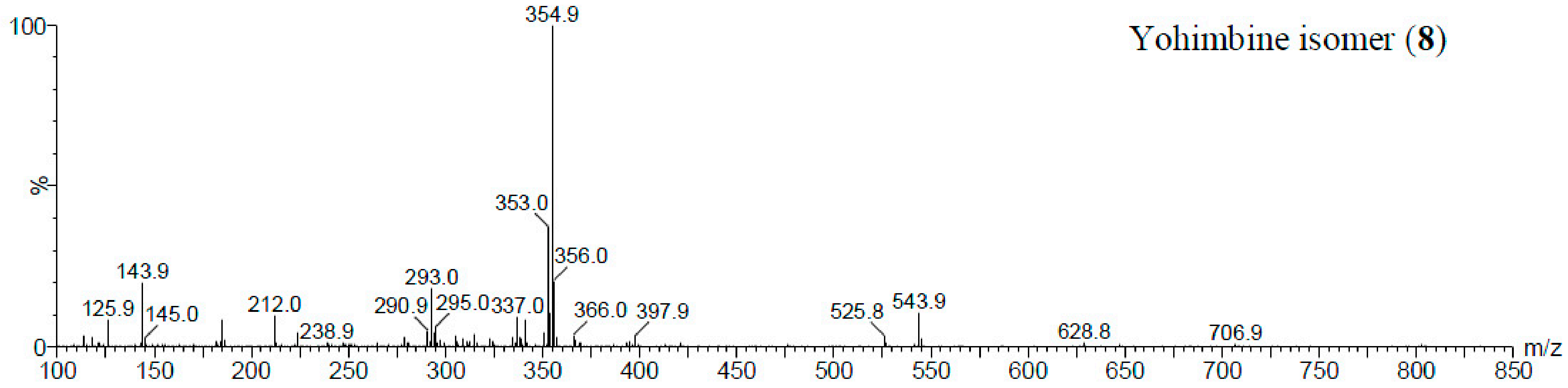

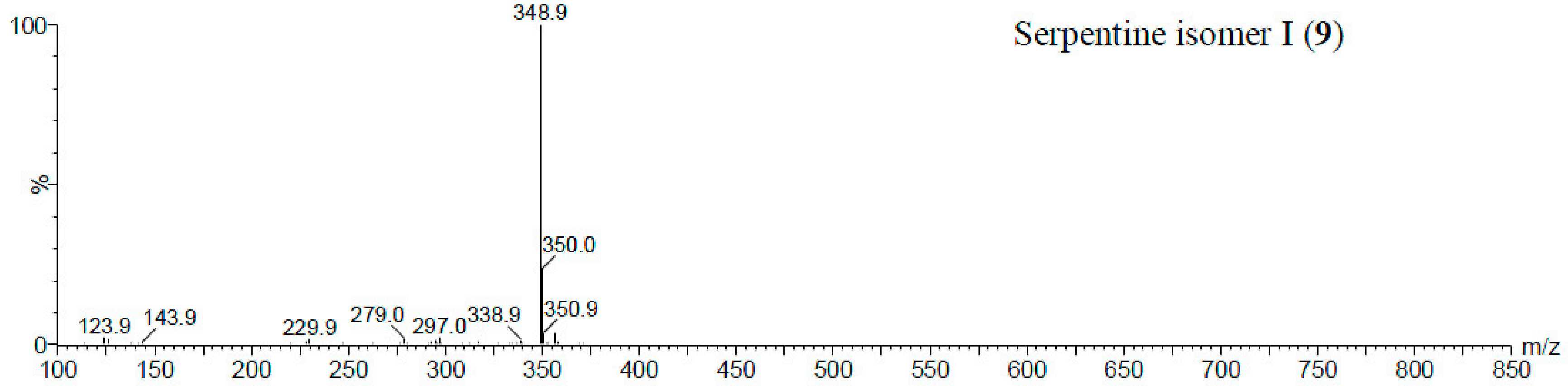

Figure S2. *Cont.*

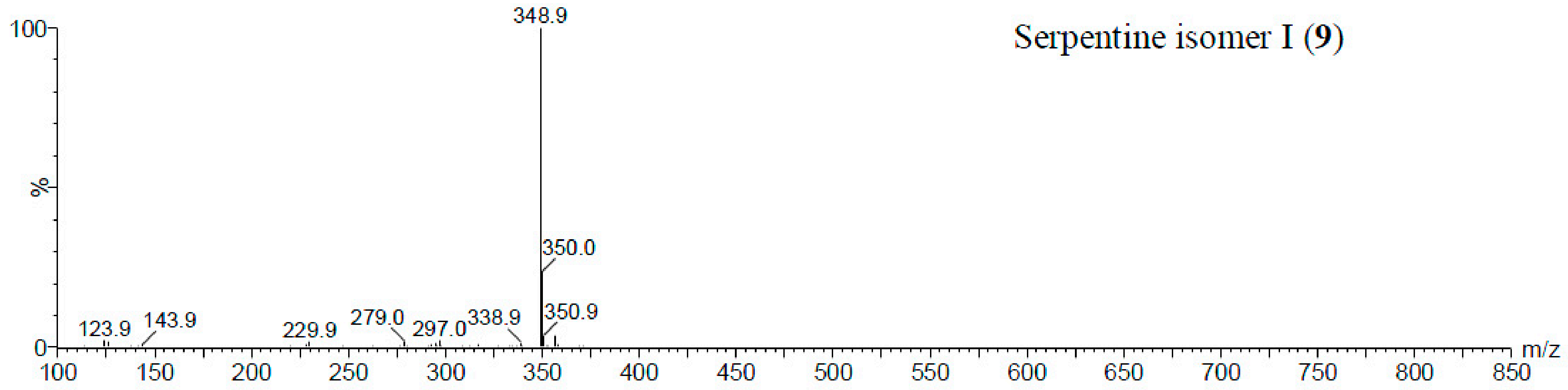

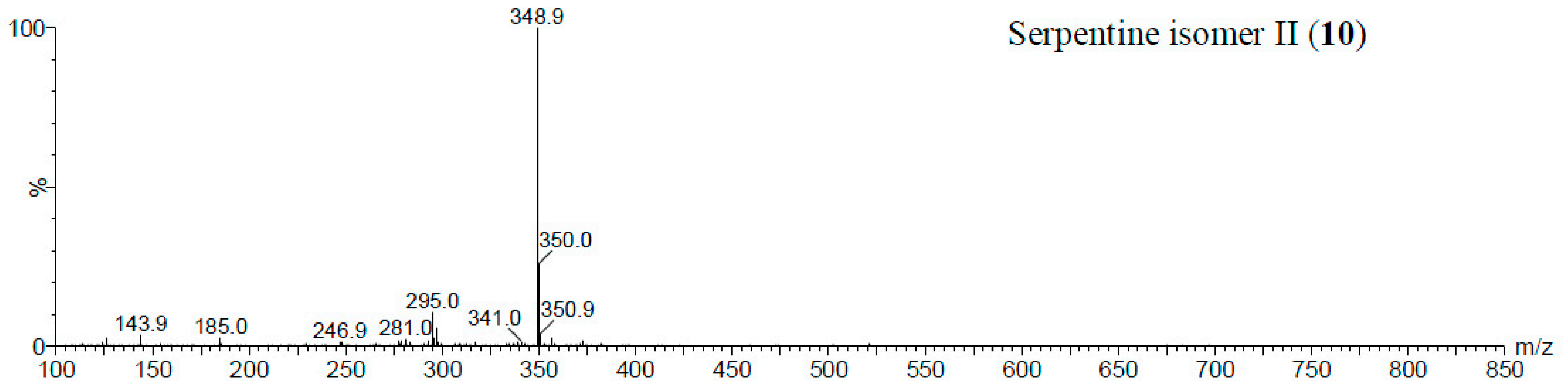

Figure S2. *Cont.*

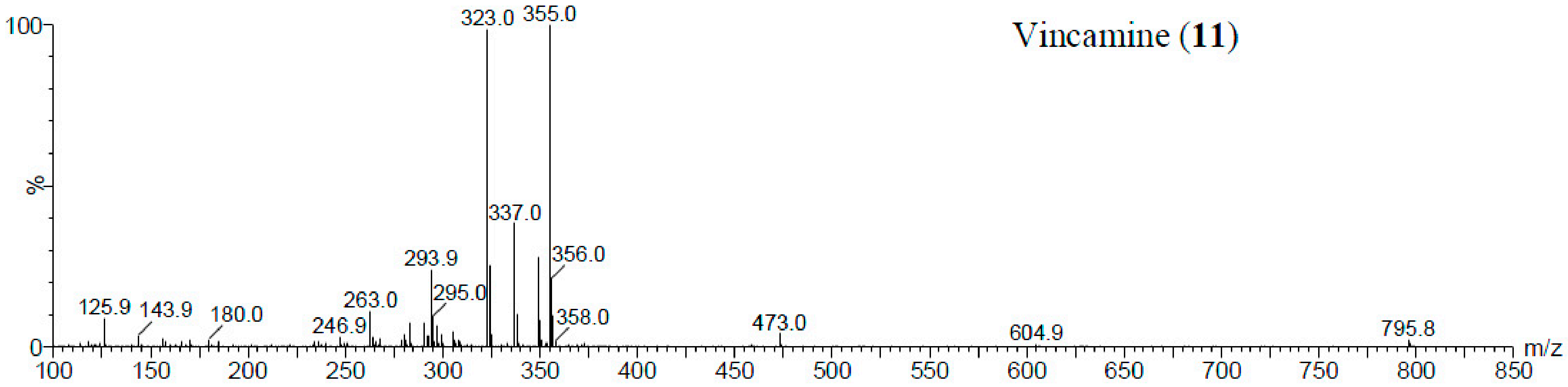

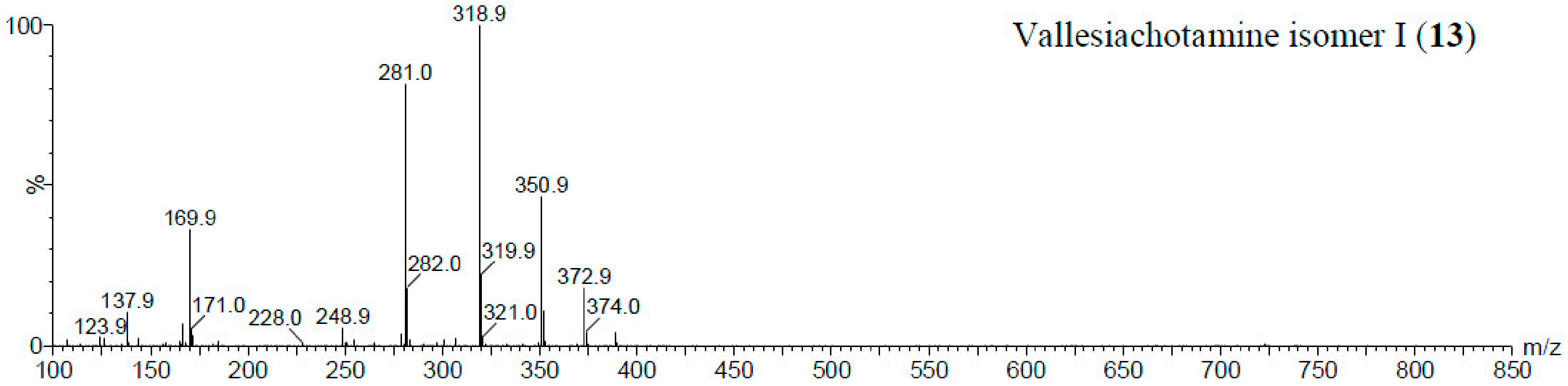

Figure S2. *Cont.*

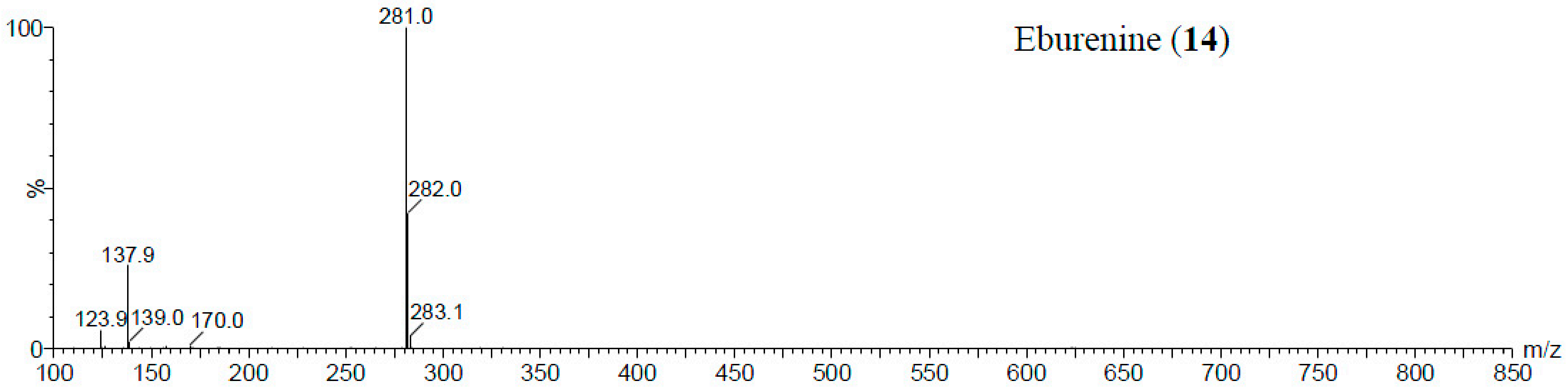

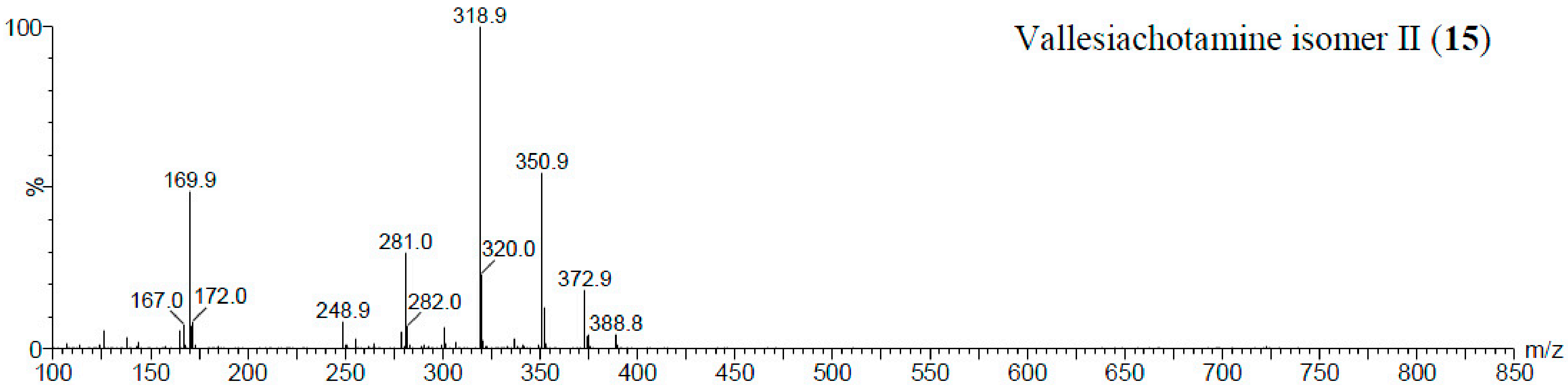

Figure S2. *Cont.*

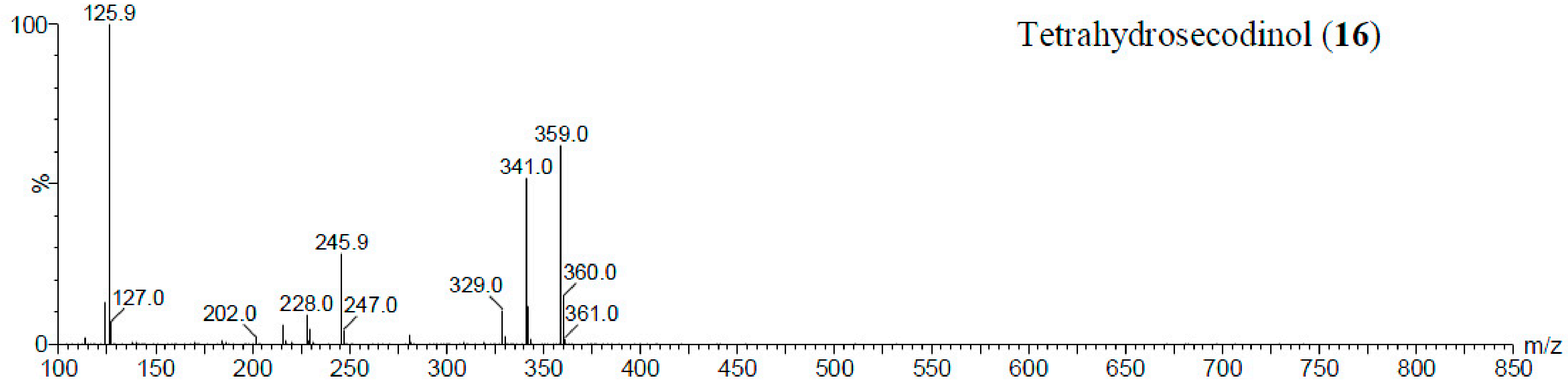

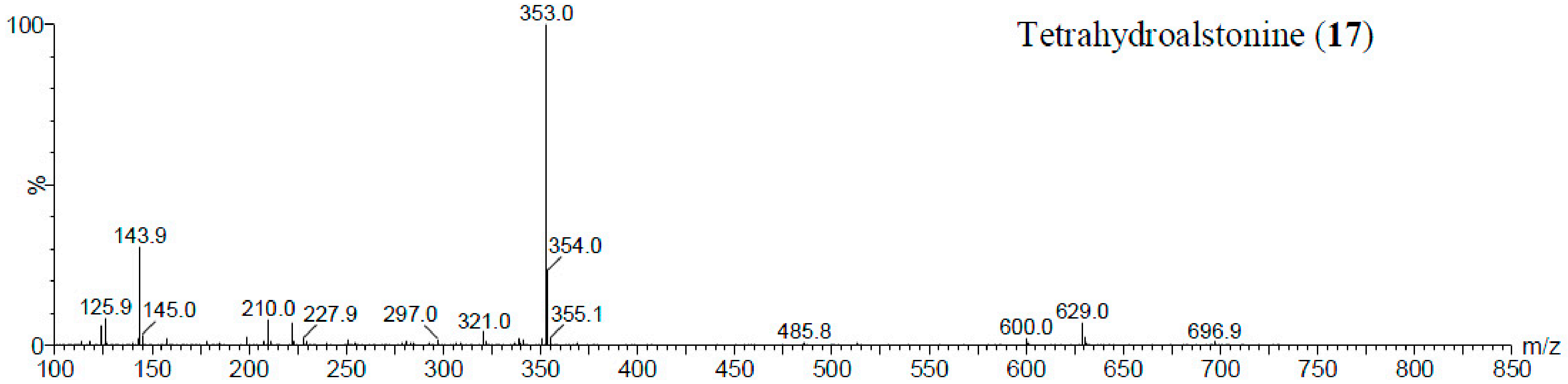

Figure S2. *Cont.*

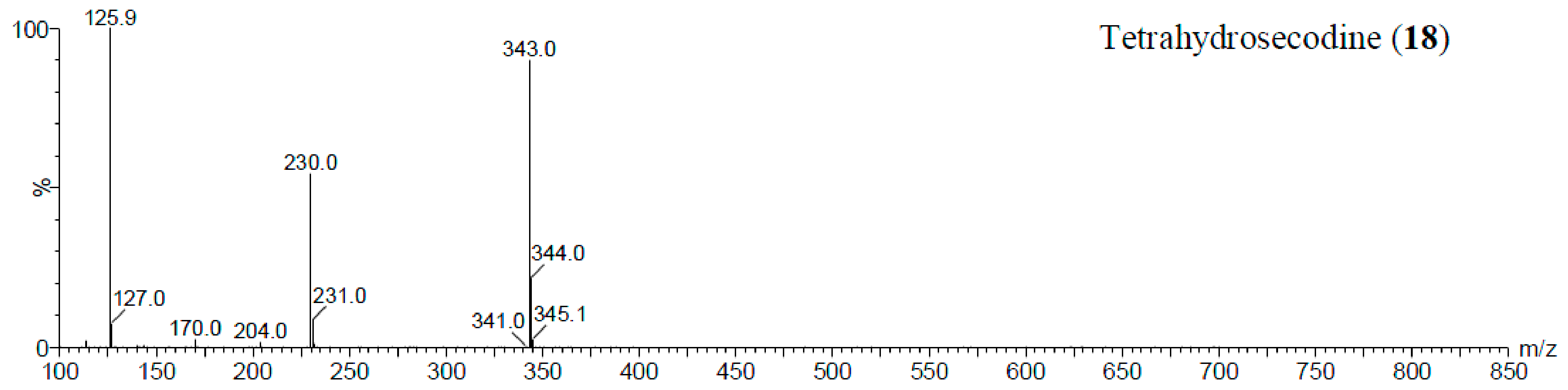

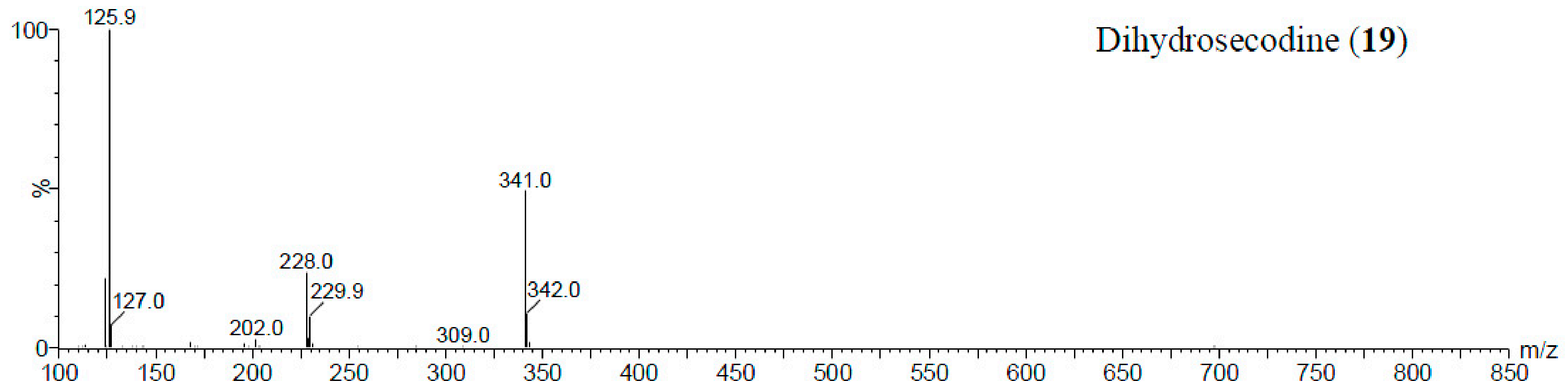

Figure S2. *Cont.*

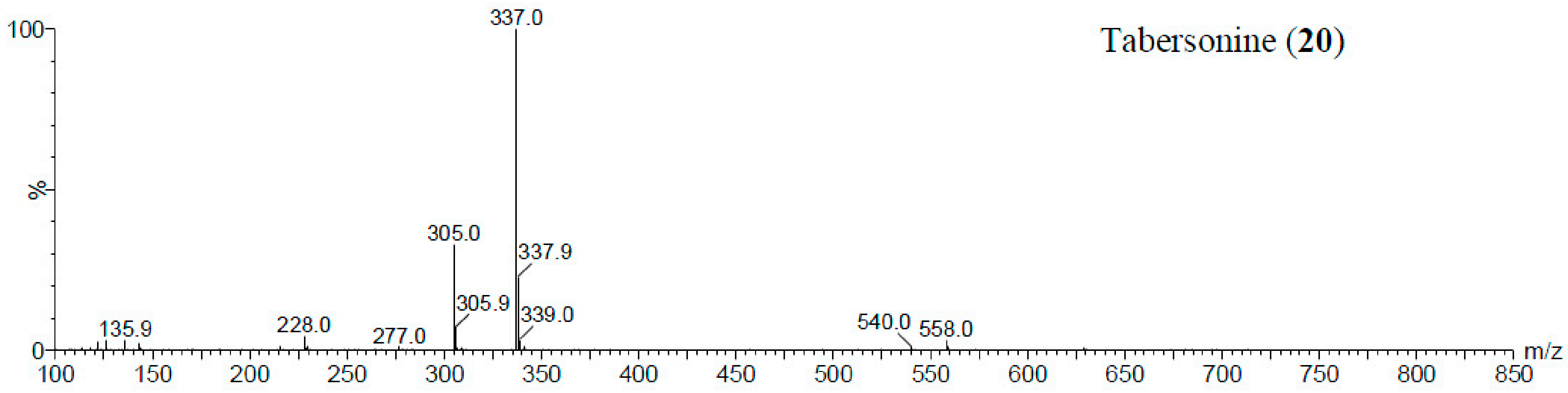

Figure S2 UPLC-MS spectra of *R. stricta* alkaloids in positive ionization mode (ESI⁻). Data for vincadifformine (**12**) is presented in Figure S3.

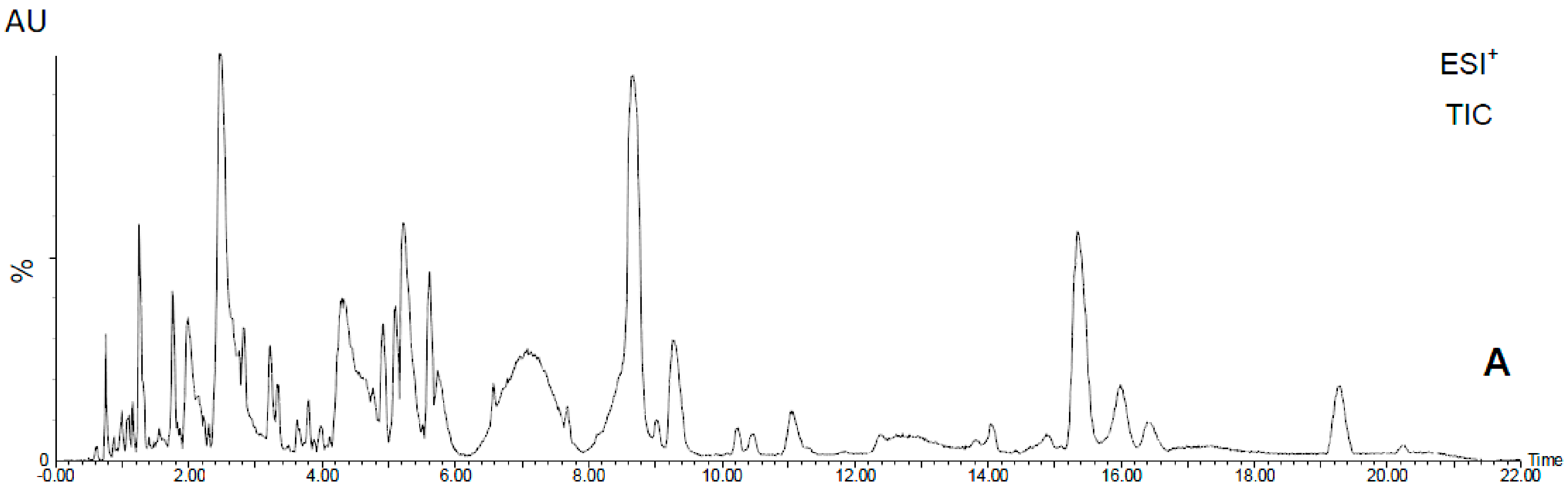

Figure S3. *Cont.*

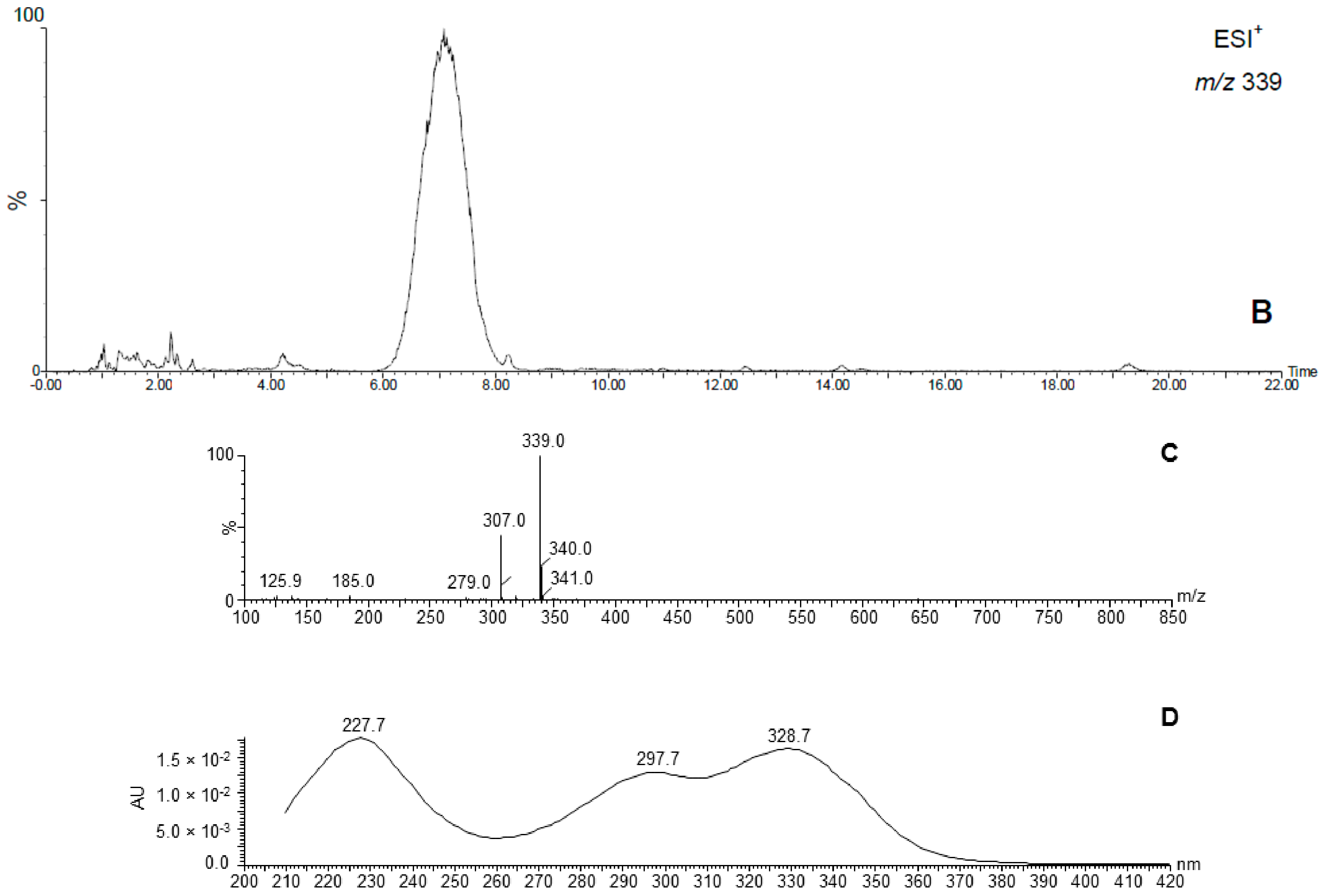

Figure S3 UPLC-PDA-MS-TIC analysis of *R. stricta* in positive ionization mode (**A**), extracted iongram of vincadifformine (**12**, channel *m/z* 339) (**B**), its UPLC-MS spectrum (**C**), and UV spectrum (**D**).
